# TTN^+^ macrophages are enriched in the human choroid plexus in Alzheimer’s disease

**DOI:** 10.1016/j.celrep.2026.117859

**Published:** 2026-08-14

**Authors:** Sagar Bhatta, Alayna Grzybowski, Gion Ortiz, Marcello DiStasio

**Affiliations:** 1 Department of Ophthalmology and Visual Science, Yale School of Medicine, New Haven, CT, USA; 2 Department of Pathology Yale School of Medicine, New Haven, CT, USA; 3 Program in Forensic Science, University of New Haven, New Haven, CT, USA; 4 Lead contact

## Abstract

The choroid plexus (ChP) regulates cerebrospinal fluid homeostasis and immune surveillance, but its cellular organization and response to Alzheimer’s disease (AD) remain incompletely defined. Here, we integrate single-nucleus RNA sequencing with spatial transcriptomics to generate a high-resolution atlas of the adult human ChP. We identify all major epithelial, stromal, and immune populations and resolve heterogeneity within macrophages, including a distinct subset expressing the giant protein TTN. These TTN^+^ macrophages exhibit coordinated cytoskeletal, stress-response, and phagocytic gene programs and localize to perivascular and stromal niches. In AD, we observe broad, cell type-specific transcriptional remodeling across epithelial, endothelial, mesenchymal, and immune compartments, with expansion of TTN^+^ macrophages and increased senescence signatures. Spatial and ligand-receptor analyses reveal altered tissue organization and macrophage interaction patterns with neighboring cells. Combined with *in situ* validations, these findings provide evidence of TTN expression by macrophages, and reveal a broader AD-associated contextual shift in human ChP immune function in which TTN^+^ macrophages are enriched.

## In brief

Bhatta et al. profile the human choroid plexus by single-nucleus RNA sequencing and spatial transcriptomics, identifying a TTN-expressing macrophage population within the stroma. These cells are expanded in donors with Alzheimer’s disease, implicating border-associated macrophages in neuroimmune changes at this CNS barrier.

## Graphical abstract

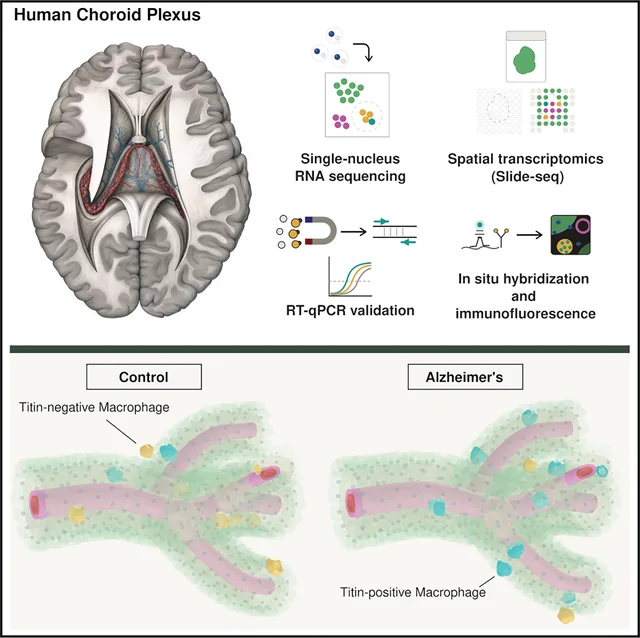

## INTRODUCTION

The choroid plexus (ChP) is a vital brain structure that consists of a network of capillaries surrounded by mesenchymal and epithelial cells and resides within the ventricles of the brain. The ChP produces cerebrospinal fluid (CSF) and, along with arachnoid membranes, forms the blood-CSF barrier. This specialized barrier and signaling hub exists at the interface between the peripheral circulation and the central nervous system (CNS). The ChP functions as an active immunological checkpoint that integrates peripheral signals with CNS-derived cues to regulate leukocyte entry, antigen presentation, and inflammatory tone.^1^ Anatomically and functionally, the ChP resides at a strategic neuroimmune nexus: resident stromal, epithelial, and endothelial cells form a scaffold for diverse immune populations that continuously survey the ventricular environment and coordinate immune responses across the blood-CSF interface.

Among these immune populations, ChP macrophages exhibit a hybrid identity. They share features with CNS border-associated macrophages (BAMs), circulating monocytes, and tissue-resident macrophages (TRMs), and display functional diversity shaped by stromal niche cues, barrier architecture, and CSF-borne cytokines.^2–4^ Despite their central role in neuroimmune communication, the molecular diversity of ChP macrophages in the adult human brain has not been fully defined, and the mechanisms that govern their migration, functional states, and integration into the ChP tissue microenvironment remain poorly understood.

Recent advances in single-cell and single-nucleus transcriptomics have begun to resolve the cellular composition of CNS border tissues and their alterations in aging and disease, revealing diverse epithelial, stromal, and immune populations with distinct molecular identities.^5–9^ These approaches have been particularly valuable for defining transcriptional heterogeneity within immune compartments, including macrophages, and for identifying cell type-specific programs associated with homeostasis and inflammation.^10^ However, key aspects of human ChP organization remain incompletely understood, including how distinct cell populations are coordinated within a shared tissue environment and how their molecular states are influenced by local context.

These challenges are especially relevant in the context of neurodegenerative disease. The ChP undergoes structural and functional remodeling in neurodegeneration, including barrier dysfunction, altered secretory activity, and immune perturbation,^11–14^ yet the molecular basis of these changes in the human ChP remains poorly defined. In particular, it is unclear how macrophage states are altered in Alzheimer’s disease (AD) and how these changes relate to broader shifts across epithelial, endothelial, and stromal compartments.

A significant gap in understanding concerns how ChP macrophages integrate mechanical signals, a dimension of immune regulation increasingly recognized across tissues.^15^ Mechanical forces shape leukocyte migration, adhesion, antigen scanning, and inflammatory activation; cytoskeletal programs are central to how immune cells sense and respond to physical cues.^16–18^ Macrophage polarization is known to be influenced by biophysical cues in the extracellular environment such as interstitial flow, stretching, spatial confinement, and matrix stiffness.^19^ How these mechanisms contribute to ChP macrophage trafficking and function are largely unknown.

To address these questions, we combined single-nucleus RNA sequencing with spatial transcriptomics to generate a high-resolution molecular atlas of the adult human ChP, enabling us to define macrophage heterogeneity and characterize coordinated, disease-associated changes across cell types.

## RESULTS

### A single-nucleus transcriptomic atlas defines the cellular landscape of the human ChP

To characterize the cellular architecture of the adult human ChP, we generated a single-nucleus RNA-sequencing (snRNA-seq) atlas from postmortem lateral ventricular ChP tissue from donors with (*n* = 4) and without (*n* = 5) AD. Integration of data across donors and technical variability was mitigated using scVI^20^ for batch correction and latent space integration, enabling robust alignment of transcriptomic signatures. Dimensionality reduction and visualization were performed with scanpy,^21^ using the integrated scVI latent space to generate high-resolution uniform manifold approximation and projection (UMAP) embeddings that revealed distinct clusters and their transcriptomic heterogeneity. Cell type annotation was performed using a custom, manually curated panel of ChP-specific marker genes (see Table S2), allowing accurate delineation of epithelial, stromal, neuronal, and immune populations.

**Figure 1.**
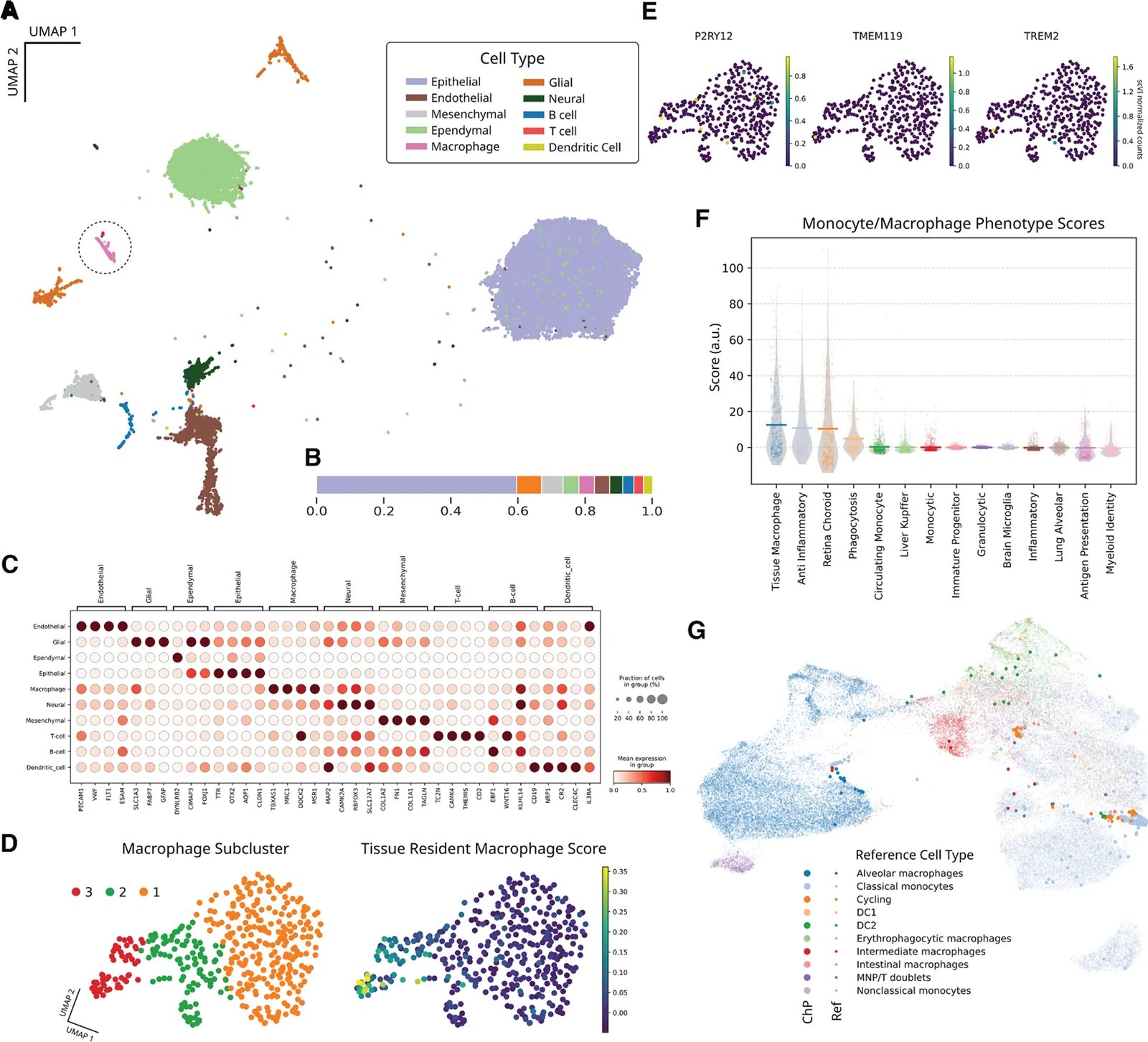
Cell type composition of human choroid plexus (A) Uniform manifold approximation and projection (UMAP) of all single-nucleus transcriptomes profiled from ChP tissue from *n* = 9 donors. Distinct clusters correspond to major choroid plexus cell types based on lineage markers, including epithelial cells, endothelial and vascular-associated cells, mesenchymal/stromal populations, immune lineages (macrophages, dendritic cells, and lymphocytes), glial and ependymal-like cells, and rare neuronal nuclei. (B) Inferred underlying proportions of each cell class, derived from intercepts of a scCODA model fit to the complete dataset accounting for compositional constraints and batch effects in the data. (C) Dot plot summarizing the expression of canonical marker genes across annotated cell populations in the choroid plexus single-nucleus RNA-seq dataset. Columns represent marker genes grouped by cell type (top), and rows correspond to annotated cell populations. Dot size indicates the fraction of cells within each population expressing a given gene; color intensity reflects the mean expression level among expressing cells. (D) UMAP visualization of macrophage subclusters and tissue-resident macrophage (TRM) scores for *n* = 7 donors for which single macrophage nuclei were recovered. Left: single-cell UMAP projection of macrophages, colored by subclusters (Cluster 1: orange; Cluster 2: green; Cluster 3: red), highlighting transcriptionally distinct populations. Right: same UMAP projection showing computed TRM score per cell. (E) UMAP plots of single cell data from macrophage cluster colored by normalized expression of microglial markers P2RY12, TMEM119, and TREM2, showing minimal expression in all macrophage subclusters. (F) Violin plots show enrichment scores for curated myeloid gene sets (see Table S3) representing monocyte and macrophage phenotypes across diverse tissues. Individual points indicate sample-level scores, with horizontal bars marking median values. (G) ChP macrophages mapped onto Cross-Tissue Immune Atlas myeloid compartment. Each dot represents an individual cell, with colors indicating different cell types; larger dots represent ChP macrophages in our current dataset.

UMAP projection of expression profiles from 37,378 nuclei revealed major epithelial, endothelial, mesenchymal, immune, glial, and neuronal compartments (Figure 1A). Bayesian compositional modeling using scCODA^22^ demonstrated that epithelial cells dominate the tissue composition (59.5%), followed by endothelial (7.4%), mesenchymal (6.4%), macrophage (4.6%), glial (4.5%), ependymal (4.7%), neural (3.9%), B cells (3.3%), dendritic cells (2.7%), and T cells (2.9%); immune lineages thus comprise ~14% of total nuclei (Figure 1B). Notably, the presence or absence of diagnosis of AD did not yield significant coefficients of any of the cell classes in the scCODA model, indicating similar proportional representation in AD as control. Canonical lineage markers were enriched within their respective cell populations with minimal cross-lineage expression (Figure 1C).

### ChP macrophages are a heterogeneous population with characteristics of BAMs

Subclustering of macrophages (*n* = 412) resolved three transcriptionally distinct groups, exhibiting differential enrichment for TRM signatures (Figure 1D). Canonical microglia-associated genes did not show high expression in any of these groups (Figure 1E). To contextualize these states, we used module scores^23^ computed in scanpy to quantify expression spanning diverse myeloid programs, including TRM identity, inflammatory activation, phagocytosis, metabolic states, and antigen presentation, which revealed substantial functional heterogeneity across clusters (Figure 1F; for a listing of genes used to define these modules, see Table S3). Mapping these cells onto a cross-tissue myeloid atlas^24^ showed that ChP macrophages occupy transcriptional positions that bridge classical monocytes, intermediate macrophages, alveolar-like macrophages, and dendritic cell states (Figure 1G), consistent with a composite peripheral-CNS border identity. The majority of the macrophages from the ChP dataset mapped to classical monocytes and cycling cells. Cells with signatures similar to alveolar macrophages, DC2 (dendritic cells), and intermediate macrophages also represented significant portions of the mapped data. Additionally, smaller subsets of cells mapped to intestinal macrophages and erythrophagocytic macrophages, highlighting the presence of gene signatures that resemble those found in these specialized macrophage types. This mapping suggests the existence of a variety of macrophage subtypes within the ChP that express gene signatures similar to immune cells in different tissues.

**Figure 2.**
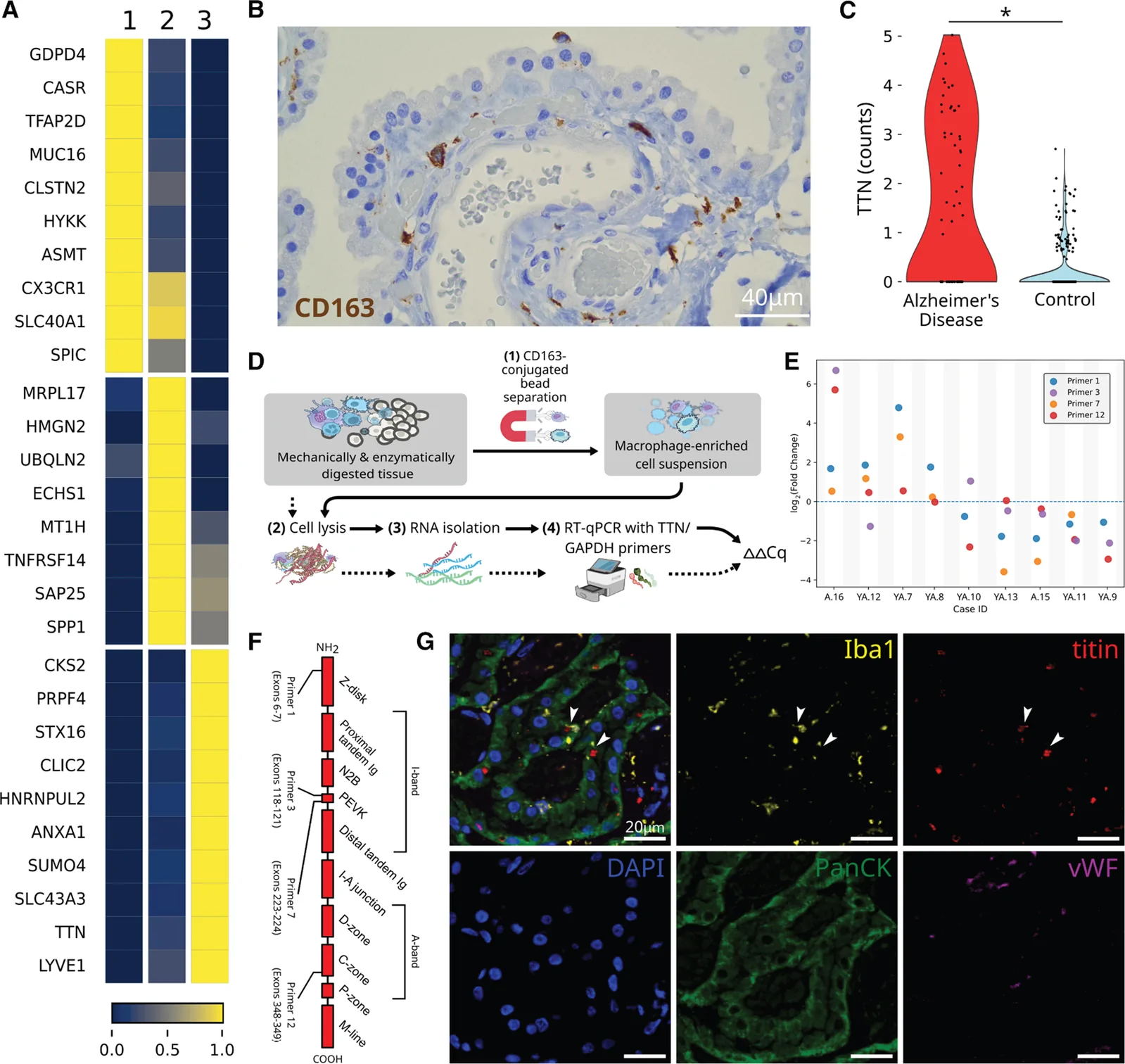
Titin-expressing macrophages are present in the choroid plexus (A) Heatmap of the top eight differentially expressed genes in each of the three ChP macrophage subclusters. Gene expression levels are represented on a normalized gradient scale. (B) DAB immunohistochemical stain of a 5 μm FFPE section of control ChP tissue using a CD163 antibody. CD163-positive macrophages are marked by brown staining, while cell nuclei are counterstained blue with hematoxylin, indicating their distribution within the vascularized ChP tissue. Scale bars, 40 μm. (C) Violin plots illustrating TTN expression levels in Alzheimer’s disease and control conditions; plot shows the distribution, density, and variability of TTN transcript counts in ChP macrophages within each condition. * = p < 0.05 (Wilcoxon rank-sum test). (D) Schematic representation of the experimental workflow for evaluation of TTN RNA enrichment in macrophages, involving tissue dissociation, CD163 positive cell enrichment, RNA isolation, cDNA synthesis, PCR amplification using isoform-specific TTN primers, and ΔΔCq calculation with GAPDH used for normalization. (E) Fold changes, computed from ΔΔCq, in TTN isoform expression relative to GAPDH in CD163-bead enriched macrophages relative to whole tissue. The *x* axis represents different primer sets (1, 3, 7, and 12), and the *y* axis shows fold changes (log scale). Each point represents a single donor sample/primer set pair. (F) Schematic representation of the TTN gene on chromosome 2 with indicated primer binding sites relative to exons. (G) Representative multiplex immunofluorescence image of ChP stained with primary antibodies for Iba1 (yellow), titin (red), Pan-cytokeratin (green), and vWF (magenta), co-stained with DAPI (blue), showing cytoplasmic titin protein in ChP macrophages (arrowheads). All scale bars, 20 μm.

Differential expression across macrophage subclusters reveals gene sets that segregate into three transcriptionally coherent modules reflecting distinct functional identities within the ChP (Figure 2A). Subcluster 1 contains signatures of barrier-associated functions such as epithelial-interaction and homeostatic tissue-residence (e.g., CX3CR1, SPIC, MUC16, CLSTN2, CASR, and HYKK). Subcluster 2 comprises macrophages enriched for tissue-resident surveillance and immune quiescence (e.g., CX3CR1, SPP1, and TNFRSF14), metabolic and mitochondrial specialization (e.g., MRPL17, ECHS1, and MT1H), and proteostatic and chromatin regulatory programs (e.g., UBQLN2, HMGN2, and SAP25), suggestive of stress-adaptive or remodeling functions consistent with long-lived, interface-associated macrophages. Subcluster 3 is enriched for genes involved in anti-inflammatory and pro-resolving activity (e.g., ANXA1 and SUMO4), tissue remodeling and matrix interaction (e.g., LYVE1 and SPP1), RNA-processing and stress adaptation (e.g., PRPF4 and HNRNPUL2), and vesicular and ion-regulatory dynamics (STX16 and CLIC2). Human ChP macrophages thus show divergent structural, metabolic, and immune states similar to those in mice^25^ and to other sites of human tissue residence.

### ChP macrophages include a titin-expressing population validated by qPCR

TTN emerged as a distinguishing transcript of ChP macrophage subcluster 3 (Figure 2A), and TTN^+^ macrophages constituted 22.6% of the total macrophage population across all cases. Immunohistochemistry with anti-CD163 antibodies confirmed the presence of macrophages embedded within the perivascular spaces and stromal and epithelial folds of the ChP in our samples (Figure 2B). Violin plots of TTN expression distributions in single macrophages demonstrated significantly higher levels in AD compared to control samples (Figure 2C). Expression of TTN RNA was evaluated in publicly available single-cell RNA-sequencing atlases, identifying TTN^+^ myeloid cells in multiple datasets (see Figures S1A and S1B). TTN expression was particularly prominent in myeloid/macrophage cells occupying adipose tissue (see Figure S1C).

To validate TTN expression independently of sequencing, we enriched for CD163^+^ macrophages using magnetic bead separation, and performed RT-qPCR for TTN transcripts in macrophage-enriched tissue and control unenriched whole ChP tissue (Figure 2D). Four distinct primer sets detected TTN transcripts at higher abundance in CD163^+^ enriched tissue compared with whole tissue in a subset of patients (Figures 2D–2F). Though the sample size precludes drawing population-level conclusions about patient characteristics associated with TTN expression in ChP macrophages from this experiment, these results support the existence of near complete TTN transcripts in macrophages occupying human ChP tissue. TTN expression was also confirmed on RT-qPCR of RNA extracted from macrophages isolated from frontal cortex, CSF, subcutaneous fat, and mesenteric fat (see Figure S2).

To determine whether titin protein was present in macrophages in the ChP, we performed multiplex immunofluorescence staining with an anti-titin antibody of postmortem human ChP samples co-stained with Iba1, a marker of macrophages/microglia, along with Pancytokeratin to stain ChP epithelial cells and vWF to delineate vascular endothelium, revealing positive staining for titin in the cytoplasm of Iba1^+^ macrophages (Figure 2G).

### TTN^+^ macrophages exhibit distinct molecular programs and occupy particular spatial niches in the ChP

**Figure 3.**
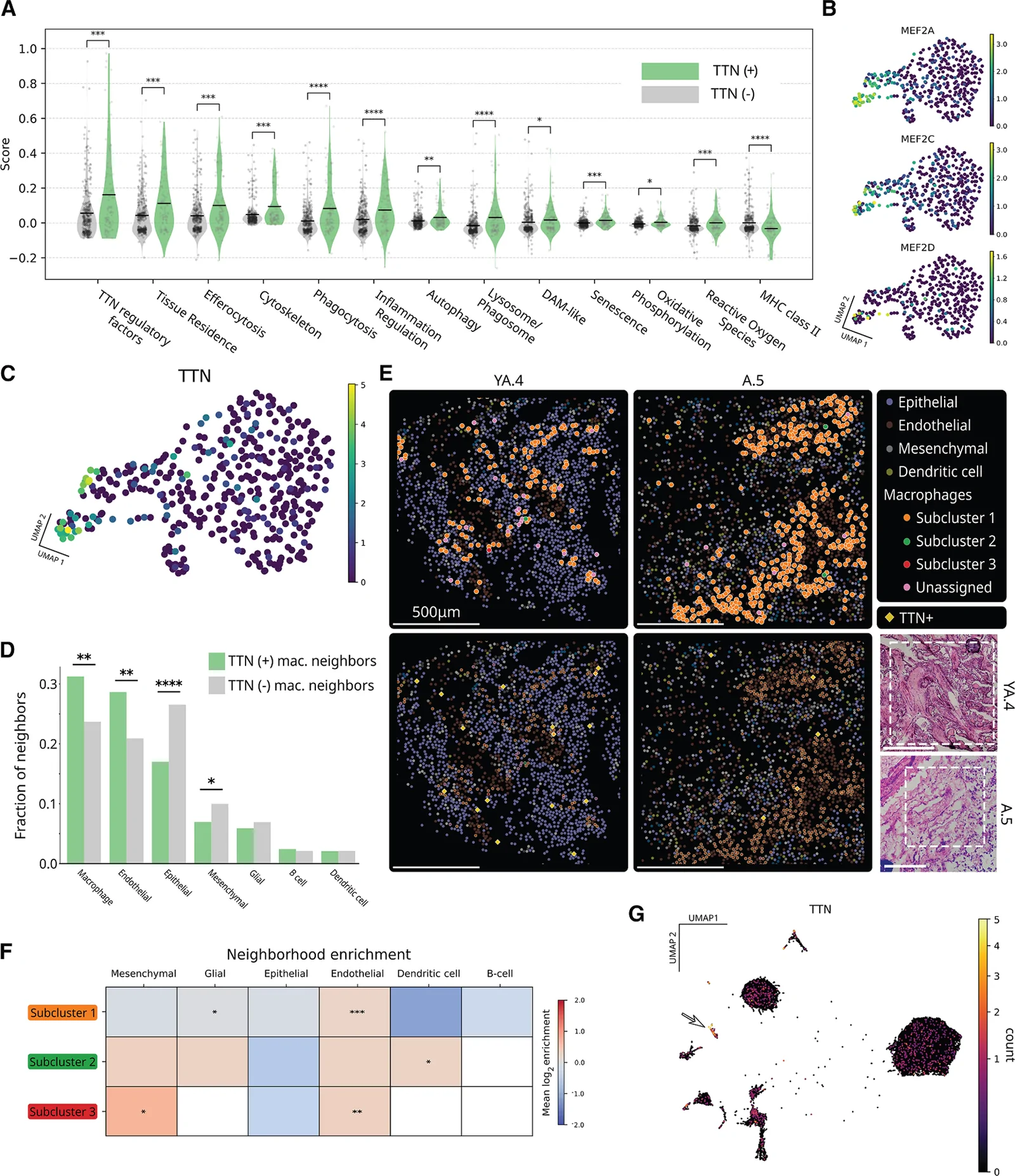
Spatial organization and functional states of TTN-expressing ChP macrophages (A) Distinct molecular programs are expressed in TTN^+^ macrophages. Violin plots showing module scores for curated macrophage gene signatures. TTN^+^ cells exhibit enhanced cytoskeletal, phagocytic, autophagy-lysosomal, inflammatory, oxidative stress-response, and MHC class II programs, along with elevated senescence and DAM-like signatures, compared with TTN^−^ macrophages. * *p* < 0.05, ** *p* < 0.01, *** p< 0.001, **** *p* < 0.0001, Wilcoxon rank-sum test. (B) MEF2 transcription factor activity in macrophage subclusters. UMAP plots of single cell data colored by normalized expression of MEF2A, MEF2C, and MEF2D highlights differential activation across macrophage populations, consistent with transcriptional profiles associated with cytoskeletal remodeling and mechanosensitive signaling. (C) UMAP plot of single cell macrophage cluster colored by TTN expression level (raw counts). (D) Altered local cellular neighborhoods of TTN^+^ macrophages. Bar plots quantifying the proportion of neighboring cell types surrounding TTN^+^ versus TTN^−^ macrophages demonstrate that TTN^+^ cells engage distinct spatial niches, with increased macrophage-endothelial and macrophage-macrophage proximity and reduced localization to epithelial and mesenchymal compartments. * *p* < 0.05, ** *p* < 0.01, **** *p* < 0.0001, Wilcoxon rank-sum test. (E) Upper row: example spatial distribution of immune and stromal populations in the ChP from two samples. High-resolution spatial transcriptomics maps showing the organization of epithelial, endothelial, mesenchymal, dendritic cell, and macrophage compartments; spots are colored according to cell type contributing predominance of transcripts. Lower row, left: TTN^+^ macrophages localize largely in perivascular space (within regions of endothelial cell enrichment), but are also present in mesenchymal compartments. Lower row, right: adjacent H&E stained sections showing reference microanatomy for spatial transcriptomics panels; corresponding areas indicated by dashed outlines. All scale bars, 500 μm. (F) Spatial neighborhood enrichment of cell types around macrophage subtypes in the choroid plexus. Heatmap showing mean log_2_ enrichment of neighboring cell types in the local spatial neighborhoods of spots containing macrophages of subclusters 1, 2, and 3 across samples (*k* = 7 nearest neighbors). Enrichment values represent the ratio of observed neighborhood frequency to global cell-type frequency within each sample, averaged across samples. Positive values indicate enrichment, while negative values indicate depletion. Statistical significance was assessed using a within-sample permutation test (2,000 permutations), in which non-macrophage cell labels were randomly reassigned while preserving spatial coordinates and macrophage identities. Empirical *p* values were computed for each sample and combined across samples using Fisher’s method. *p < 0.05, **p < 0.01, ***p < 0.001. (G) UMAP plot of complete single cell dataset showing TTN expression (raw counts). Arrow indicates macrophage cluster (see Figure 1A for all cell type labels).

We next asked whether TTN expression defines a functionally distinct macrophage state. Gene signature scoring^21,23^ revealed that TTN^+^ cells display coordinated upregulation of cytoskeletal remodeling, phagocytic, lysosomal, autophagy, inflammatory, and oxidative stress-response pathways, as well as elevated DAM-like and senescence signatures (Figure 3A; gene lists for modules are given in Table S5). Given the close link between TTN and mechanotransduction, we examined expression of MEF2A, MEF2C, and MEF2D, transcription factors with known roles in cytoskeletal regulation and stress adaptation. All three showed enrichment in the TTN^+^ macrophage subcluster 3 (Figures 3B and 3C), supporting a possible TTN-MEF2 axis in ChP macrophages. These patterns suggest that TTN^+^ macrophages occupy a mechanically and metabolically engaged phenotype.

Spatial transcriptomics using Slide-seq^26^ on ChP from four donors with AD, with integration of our snRNA-seq data via GIMVI,^27^ revealed that TTN^+^ macrophages preferentially cluster near other macrophages and show reduced proximity to epithelial and mesenchymal cells relative to TTN^−^ cells (Figure 3D). Spatial transcriptomic mapping revealed a structured organization of immune and stromal compartments within the ChP, consistent with its folded epithelial architecture surrounding a vascularized core (Figure 3E). Epithelial cell-enriched regions formed contiguous layers, while endothelial and mesenchymal populations localized to stromal interiors, delineating perivascular niches. Within this spatial framework, macrophages were distributed throughout the tissue but exhibited non-random localization patterns. TTN^+^ macrophages were preferentially enriched in perivascular regions characterized by high endothelial cell density, with additional presence extending into adjacent mesenchymal compartments. This distribution contrasts with a more diffuse localization of TTN^−^ macrophages, and suggests that TTN expression is associated with positioning in mechanically and functionally distinct microenvironments. The spatial correspondence between TTN^+^ macrophages and vascularstromal interfaces supports a model in which these cells are specialized for interactions within dynamic, shear-, and tension-exposed regions of the ChP.

Spatial neighborhood analysis around macrophage subtypes revealed distinct cell type enrichment patterns (Figure 3F). For each sample, spatial neighborhoods were defined using *k*-nearest neighbors (*k* = 7), and the probability of observing each non-macrophage cell type in the local vicinity of spots containing macrophages of subclusters 1, 2, and 3 was computed relative to the global cellular composition of the sample. This analysis identified significant enrichment of mesenchymal and endothelial neighbors for the TTN-enriched subcluster 3, compared to enrichment of endothelial neighbors only for subcluster 1, and an enrichment for dendritic cell neighbors of macrophage subcluster 2.

**Figure 4.**
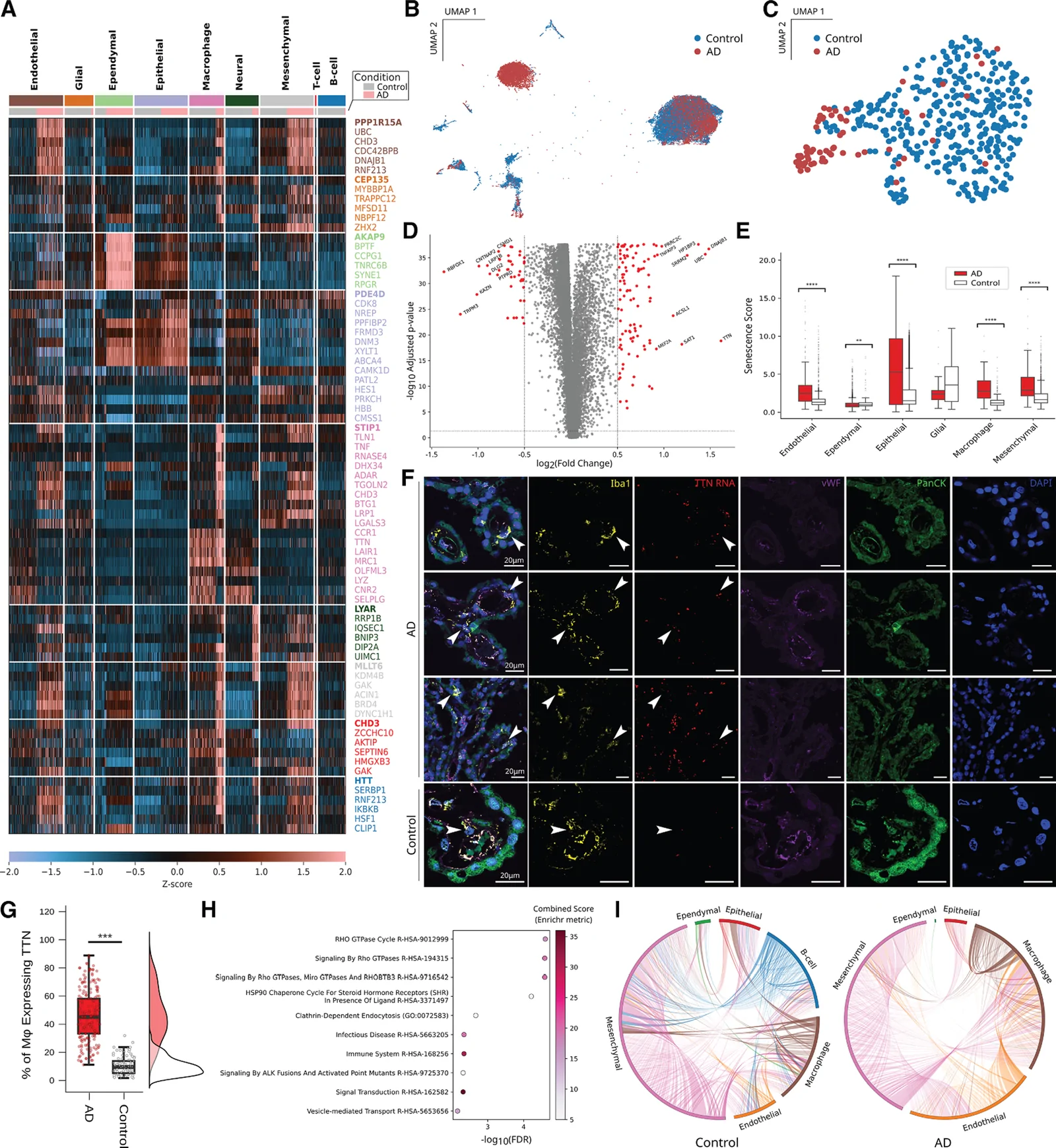
Alzheimer’s disease reshapes transcriptional state, senescence programs, and interaction networks of choroid plexus macrophages (A) Cell type-resolved heatmap of AD-associated gene expression in human choroid plexus. Single-cell RNA sequencing data are shown as a heatmap of gene expression across individual cells, grouped by cell class and disease condition. Expression values are *Z* scored per gene across all cells for visualization. Columns represent individual cells, ordered by cell type and condition (control vs. AD), with top annotation bars indicating cell class (upper) and condition (lower). Rows represent genes, including the top differentially expressed genes upregulated in AD within each cell class, supplemented with selected genes of interest. Within each cell class, genes are ordered by their relative enrichment in AD compared to control cells. (B) UMAP of all single-nucleus transcriptomes profiled from ChP tissue, colored by condition. (C) UMAP of single-nucleus transcriptomes profiled from ChP macrophages, colored by condition. (D) Global transcriptional changes in choroid plexus macrophages in Alzheimer’s disease (AD). Volcano plot depicting differentially expressed genes (DEGs) between AD and control choroid plexus macrophages. For each gene, the fold change (*x* axis) is plotted against the −log_10_ adjusted *p* value (*y* axis). Genes surpassing the significance thresholds (adjusted *p* value < 0.05 and |log_2_FC| > 0.5) are highlighted in red, while dashed lines denote the statistical and fold-change cutoffs. Representative genes with the largest effect sizes or strongest statistical significance are annotated. (E) Senescence scores derived using the SenePy framework demonstrate increased senescence signatures across multiple choroid plexus cell types, including macrophages (** p < 0.01, **** *p* < 0.0001, Wilcoxon rank-sum test). (F) Example micrographs of AD and control choroid plexus, stained with immunofluorescence markers Iba1 (yellow), Pan-cytokeratin (green), and vWF (magenta), co-stained with DAPI (blue), along with RNAscope single molecule FISH probes for TTN RNA (red). Arrowheads indicate Iba1^+^ macrophages containing TTN RNA. All scale bars, 20 μm. (G) Frequency of macrophages containing TTN transcripts in RNAscope assay, quantified as percentage of total number of macrophages present per field. An increased fraction of TTN-expressing macrophages are found in AD ChP compared to control (*** = P(AD > control) > 0.999, Bayesian independent samples *t* test reporting posterior probability). (H) Enrichr over-representation analysis performed using across GO Biological Process (2023), KEGG (2021), and Reactome (2022) gene sets. The dot plot summarizes the top enriched pathways, with the *x* axis representing enrichment significance (−log_10_ adjusted *p* value) and dot color reflecting the Enrichr combined score integrating statistical significance and deviation from background expectation. Pathways are ranked by adjusted *p* value, highlighting the major biological processes associated with the observed gene expression changes. (I) Circos plots of ligand-receptor interactions inferred by LIANA show altered macrophage connectivity in AD. Sectors represent individual cell types, and links indicate inferred signaling interactions from ligand-expressing source cells to receptor-expressing target cells. Link width, opacity, and radial prominence are scaled according to interaction strength (LIANA scaled weight), with larger and darker links denoting stronger predicted communication. Links are colored according to source cell type. Sector size is proportional to the cumulative interaction strength associated with each cell type. Cell types with no interactions crossing significance threshold *p* < 0.05 are omitted from the plot.

Global analysis of TTN transcript counts across cell types revealed high expression in macrophages accompanied by broad low expression in other cell types (Figure 3G). Single-nucleus RNA sequencing preferentially recovers nuclear transcripts and under-detects exceptionally long genes such as TTN, so the cross-cell type comparison in Figure 3G reflects relative nuclear transcript abundance rather than total cellular TTN. Consistent with this, *in situ* hybridization revealed abundant epithelial TTN RNA (Figure 4F), greater than the single-nucleus data alone would indicate, while titin protein was present at more modest levels in epithelium by immunofluorescence (Figure 2G): a transcript-protein relationship consistent with post-transcriptional regulation. TTN is therefore expressed broadly across ChP compartments and is not restricted to macrophages.

### AD drives transcriptional remodeling, senescence, and alteration of interaction networks in ChP macrophages

To define how AD reshapes transcriptional programs across ChP cell populations, we examined cell type-resolved gene expression patterns in our single-nucleus RNA transcription data. Visualization of differentially expressed genes across all major cell classes revealed widespread but structured transcriptional remodeling in AD.

AD-associated changes are organized into coherent gene expression blocks within specific cell types rather than reflecting global expression shifts (Figure 4A). In addition to shifts in macrophages, other cell populations also exhibited disease-associated transcriptional changes. Epithelial and ependymal cells showed alterations in genes related to cellular stress, transport, and barrier-associated functions, while mesenchymal and endothelial compartments demonstrated modulation of genes linked to extracellular matrix organization and vascular signaling. Neuronal and glial populations exhibited more subtle but detectable shifts.

A UMAP plot of the cells shows contributions to all major cell type classes from both AD and control samples, with disease-associated within-class shifts (Figure 4B); corresponding to the lack of significant disease-condition coefficients for any of the cell classes in the scCODA composition model. Macrophage subcluster composition differed between conditions, with control samples predominantly composed of subcluster 1 (71% versus 13% in AD), whereas AD samples were enriched for subcluster 3 (73% versus 3% in controls), with more modest shifts observed in subcluster 2 (χ^2^ test, *p* < 1 × 10^−17^; Figure 4C). The presence of elevated proportions of TTN^+^ macrophages was seen in all donors with AD from which macrophages were recovered (see Table S4).

We performed differential expression analysis between AD (*n* = 63) and control (*n* = 349) macrophages. AD macrophages exhibited a broad transcriptional shift, with TTN, STAB1, SAT1, MEF2A, and TNFAIP3 among the most significantly upregulated genes, while neuronal-adhesion and cytoskeletal-associated transcripts (RBFOX1, PTPRD, DLG2, and CSMD1) were prominently downregulated (Figure 4D). These changes highlight disrupted cytoskeletal, stress-response, and immune-modulatory programs in AD macrophages.

Because many TTN-associated pathways intersect with cellular aging, we also quantified senescence scores across ChP cell types. Senescence scores were elevated across multiple cell types in AD, including macrophages, epithelial, and endothelial compartments, with increases of similar magnitude across these populations (Figure 4E).

TTN RNAscope with Iba1/Pan-cytokeratin/vWF immunofluorescence imaging identified TTN transcripts in both nuclei and cytoplasm of Iba1^+^ cells (Figure 4F). Quantitative analysis of Iba1^+^ cells indicated that TTN^+^ macrophages were present in control ChP but substantially expanded in AD tissue (Figure 4G), validating the transcriptomic findings of disease-associated expansion of the TTN^+^ macrophage population.

Pathway enrichment analysis (Figure 4H) strongly implicated dysregulation of Rho family GTPase signaling, a core regulatory axis that integrates mechanical inputs with cytoskeletal remodeling, adhesion dynamics, and migratory behavior. RhoA, Rac1, and Cdc42 act as molecular switches that coordinate actin polymerization, membrane protrusion, and contractility. These programs are essential for macrophage motility, phagocytosis, and environmental sensing, consistent with altered mechanotransduction and responsiveness to tissue cues. Additional perturbations are seen in clathrin-dependent endocytosis, HSP-90 chaperone activity, vesicle trafficking, and immune activation pathways, consistent with remodeled environmental responsiveness.

To identify upstream regulatory programs underlying transcriptional remodeling in AD, we performed SCENIC-based regulon analysis in ChP macrophages (see Table S6). This revealed a set of transcription factor networks with increased activity AD relative to controls, including AP-1 family members (JUN, JUNB), RUNX (RUNX1), and chromatin- and stress-associated regulators such as HMGB1 and HMGB2. Additional enrichment was observed for transcription factors linked to calcium signaling and cytoskeletal dynamics, including NFATC2, as well as factors associated with stress adaptation and metabolic regulation, such as FOXO3 and CLOCK. Notably, several of these regulons showed consistent increases across macrophage subtypes, indicating that regulatory changes are not restricted to a single subpopulation but instead reflect a broader shift in macrophage state. In contrast, regulators associated with canonical developmental or lineage-specifying pathways, including Wnt/β-catenin-related factors (TCF12 and TCF7L2), exhibited more modest or variable changes. Overall, these findings indicate that AD-associated transcriptional remodeling in ChP macrophages is accompanied by coordinated activation of stress-responsive, chromatin-associated, and cytoskeleton-linked regulatory programs.

To quantify changes in cell-cell communication in AD, we performed ligand-receptor inference using LIANA^28^ and compared interaction patterns across cell types (Figure 4I). Globally, the number of retained interactions increased in AD (581 vs. 501 in controls). Focusing on macrophage interactions with other cell types, we observed a shift in both magnitude and directionality. Outgoing interactions from macrophages increased modestly in AD (44 vs. 26 edges; log_2_FC = 0.76), whereas incoming interactions to macrophages increased more substantially (63 vs. 28 edges; log_2_FC = 1.17). A similar pattern was observed for interaction strength, with a relatively modest increase in macrophage outgoing signaling (log_2_FC = 0.44) compared to a larger increase in incoming signaling (log_2_FC = 1.33). At the level of specific cell-cell pairs, macrophage interactions exhibited partner-specific remodeling. Outgoing interactions from macrophages were most increased toward other macrophages, but decreased toward other cell types. In contrast, incoming interactions to macrophages were broadly increased across multiple compartments, with the largest gains arising from endothelial and mesenchymal cells.

## DISCUSSION

The ChP is increasingly recognized as a dynamic neuroimmune interface whose cellular states adapt to age, injury, and neurodegeneration. ChP macrophages are a mixed population of long-lived, yolk sac-derived macrophages and replenishing bone marrow-derived macrophages that are adapted to the epithelial-stromal interface, positioned at the front line of blood-CSF communication.^5,8^ In this study, we identified a transcriptionally diverse macrophage compartment within the human ChP. Cross-tissue atlas mapping positions these cells at the intersection of TRM programs, monocyte-like states, and stress-responsive immune signatures. These findings align with previous descriptions of BAM heterogeneity and niche-driven specialization, while highlighting a range of phenotypic states in human ChP macrophages. The background stromal, epithelial, and endothelial cell milieu represents a unique structural context in which ChP macrophages operate, supporting immune sampling, antigen presentation, and barrier-associated surveillance.

### ChP macrophages show specialization for their tissue microenvironment

By combining single-nucleus and spatial transcriptomics, our study provides a comprehensive transcriptional and spatial atlas of macrophages in the adult human ChP. BAMs, of which ChP macrophages are a specialized subset, are myeloid cells that reside at CNS interfaces, including the meninges and perivascular spaces, where they form a distinct compartment from parenchymal microglia.^29^ BAMs are defined by transcriptional programs that integrate immune surveillance, antigen presentation, and regulation of leukocyte trafficking.^6,8^ Developmentally, many BAM populations arise from embryonic progenitors and are maintained through local self-renewal,^3^ although monocyte-derived cells contribute under inflammatory or disease conditions, and in homeostasis in the ChP.^5^ Blood-derived macrophages have been demonstrated to play a critical role in CNS tissue repair after injury, mediated in part by IL-10.^30,31^ Functionally, BAMs coordinate communication between the peripheral immune system and the CNS, shaping immune entry, inflammatory tone, and tissue homeostasis at neurovascular interfaces, and are increasingly implicated in neurodegenerative and neuroinflammatory diseases.

Among ChP macrophages, three distinct subsets have been described in mice: an Slc40a1^+^ subset located near larger vessels co-expressing Spic and Clec4n; a subset of Lyve1^+^ cells found on the epiplexus surface; and an Spp1-expressing subset.^25^ We find human subsets overlapping with the three seen in mice. Overlapping with the LYVE1^+^ subset, our data reveal an unexpected mechanobiological program centered on titin.

The discovery of TTN-expressing (TTN^+^) macrophages in the human ChP, and their selective expansion and molecular reprogramming in AD, defines a previously unrecognized axis of macrophage mechanotransduction at the brain’s borders.

### Titin expression identifies a distinct macrophage state

TTN is canonically understood as a structural and elastic scaffold that governs sarcomere mechanics in striated muscle. However, emerging work in lymphocytes demonstrates that immune cells can deploy TTN isoforms to regulate membrane tension, adhesion, and migratory behavior.^32,33^ As in other leukocytes,^34^ these functions depend on dynamically tuning actin architecture in response to mechanical cues.^35^ TTN-deficient lymphocytes display impaired motility and altered actin dynamics, identifying TTN as a master regulator of immune cell trafficking. These observations suggest that TTN operates as a mechanotransductive scaffold that integrates cytoskeletal tension, cell-environment interactions, and signal responsiveness; these processes are central to immune cell behavior within mechanically heterogeneous tissues like the ChP.

The primer sets we used in PCR validation (see Figure 2F) in CD163-expressing macrophages target non-overlapping regions of the TTN transcript including the Z-disk region, the I-band region (PEVK), and the A-band region (C-zone super repeats), indicating expression of extended TTN isoforms rather than short or fragmented transcripts, and suggesting that macrophages express structurally complex TTN variants. This, together with *in situ* histologic detection, establishes TTN as a feature of a macrophage subset in the human ChP, extending TTN biology beyond lymphocyte trafficking and adding to our understanding of immune mechanobiology.

Interpreting TTN across ChP cell types requires accounting for both the transcript and the assay. TTN is the largest gene in the human genome, and single-nucleus RNA sequencing, which samples nuclear RNA and under-recovers very long transcripts, is expected to underrepresent total cellular TTN. Our *in situ* data bear this out. Epithelial cells show abundant TTN RNA by RNAscope alongside the low per-nucleus epithelial TTN measured by sequencing, indicating that the single-nucleus values reflect relative nuclear abundance rather than absolute expression. Epithelial titin protein was detected by immunofluorescence at more modest levels than the transcript, consistent with post-transcriptional regulation in these cells. We therefore do not interpret TTN as macrophage-restricted. Rather, TTN is expressed across ChP compartments, and our data identify a specific macrophage subset in which TTN marks a coordinated cytoskeletal, phagocytic, and stress-response program that expands in AD. Interestingly, spatial transcriptomic analysis identified enrichment of the TTN^+^ macrophage population localized near endothelial cells, suggesting a preferential perivascular distribution and histologic studies identified TTN^+^ macrophages in this location.

TTN^+^ cells show heterogeneous but elevated expression of MEF2A, MEF2C, and MEF2D, transcription factors activated downstream of cytoskeletal deformation, Ca^2+^ flux, and RhoA signaling.^15,36^ In proinflammatory conditions, MEF2C contributes to classical activation in macrophages,^37^ in contrast to brain microglia, in which it mitigates proinflammatory TNF and IL-1β production.^38^ Consistent with this, our data show that TTN^+^ ChP macrophages display elevated MEF2A/C/D activity and upregulation of pathways involved in cytoskeletal remodeling, phagocytosis, oxidative stress responses, MHC class II antigen presentation, and autophagy. In the densely folded, fluid-exposed, and mechanically active environment of the ChP, TTN expression may therefore provide a structural and regulatory advantage for macrophages navigating stromal surfaces, engaging epithelial barriers, and responding to blood and CSF flow-derived shear forces.

Like other BAMs, ChP macrophages exhibit specialized morphologies and dynamic processes shaped by the mechanical and biochemical attributes of their niche.^39^ TTN expression provides a plausible molecular mechanism for coordinating these specialized cytoskeletal behaviors. The presence of TTN^+^ macrophages fills a gap in our understanding of how ChP macrophages sense and respond to mechanical forces within the epithelial-stromal microenvironment. In lymphocytes, TTN isoforms regulate microvilli formation, integrin-mediated adhesion, and mechanical properties that enable trafficking.^32^ Similarly, the TTN^+^ macrophage population identified here is enriched for transcriptional programs linked to cytoskeletal remodeling and adhesion, and is preferentially localized to vascular-adjacent niches, supporting a model in which TTN contributes to organizing structural and functional states across immune cell types.

### Signaling interactions with titin in cytoskeletal control and environmental sensing

Titin has well-characterized interactions with actin and actin-associated complexes, including binding via its PEVK region and Ig-like domains, that enable it to modulate cytoskeletal tension and contribute to force transmission and mechanosensing in cytoskeletal networks under load.^40,41^ In lymphocytes, titin is required for mediation of chemokine signaling by RhoA and Rac1.^32^ In macrophages, TTN expression may similarly tune the sensitivity of Rho-actin circuits, with downstream effects on force generation, motility, and immune synapse formation.

Differential expression and pathway enrichment analyses revealed substantial perturbation of Rho-family small GTPase pathways in AD macrophages. RhoA, Rac1, and Cdc42 orchestrate actin polymerization, contractility, and membrane dynamics that enable leukocyte migration, adhesion, and phagocytosis.^42–44^ Prior studies demonstrate that Rho-GTPase signaling controls these functions in macrophages^42,45–47^ and interfaces with inflammatory signaling including NF-κB and oxidative stress responses, representing a potential link from mechanical inputs to immune function.

In this framework, TTN^+^ macrophages may be more responsive to environmental cues as they navigate the biomechanically complex epithelial folds, fluid shear forces, and variable substrate stiffness of the ChP. The observation that TTN^+^ macrophages occupy spatial niches enriched for endothelial interfaces suggests that they reside at boundaries where vascular, stromal, and epithelial compartments intersect, which may expose these cells to localized hemodynamically driven gradients in shear, tissue deformation, and substrate stiffness that could engage titin-associated mechanotransduction pathways.

### A disease-associated program emerges in AD ChP macrophages

AD is increasingly recognized as a disorder involving complex neuroimmune imbalances.^48–51^ While microglial activation in the parenchyma has been extensively studied,^52,53^ the ChP represents an overlooked interface where peripheral immune cells interact with CNS-derived signals.^1,13,14^ The ChP epithelium and its resident macrophages orchestrate leukocyte trafficking into cerebrospinal fluid and regulate inflammatory tone throughout the brain.^11,54,55^

The expansion of TTN^+^ macrophages in AD suggests that this mechanosensitive program becomes preferentially induced or selected under disease conditions. AD macrophages exhibit coordinated upregulation of TTN itself and MEF2-linked signatures, along with Rho-GTPase pathway dysregulation, oxidative stress responses, and hallmarks of cellular senescence. These changes are accompanied by marked downregulation of adhesion- and cytoskeleton-associated transcripts such as RBFOX1, PTPRD, DLG2, and CSMD1, consistent with altered structural and signaling capacity.

Our analysis of ligand-receptor interactions in AD indicates that intercellular signaling networks are not uniformly increased or diminished but instead undergo directional and cell type-specific remodeling. In particular, macrophages exhibit a disproportionate increase in incoming interactions from endothelial, mesenchymal, and epithelial compartments, alongside more modest and heterogeneous changes in outgoing signaling. This shift suggests that macrophages in AD may become more responsive to environmental cues while altering their signaling outputs to specific partners. One interpretation is that increased incoming signaling reflects heightened integration of these cells within the local microenvironment, particularly at vascular and stromal interfaces where mechanical and inflammatory cues converge.

These findings support a model in which AD-associated macrophage reprogramming, potentially in macrophages recently recruited to the ChP, reflects coordinated changes in transcriptional state and network connectivity, resulting in a population that is both more environmentally responsive and functionally specialized within the ChP niche.

The relationship between TTN^+^ macrophage abundance and AD severity could not be assessed in the present study, as datasets included only a single donor with intermediate-level AD neuropathologic change. While donor-level analyses across the broad cohort demonstrate consistent enrichment of TTN^+^ macrophages in AD, future studies incorporating larger, severity-stratified cohorts with matched spatial and transcriptomic profiling will be required to determine whether these changes scale with disease progression.

### Implications for neuroimmune biology and future directions

This study provides single cell and spatial transcriptomic atlases of adult human ChP from subjects with and without AD, a resource for future studies of the blood-CSF barrier in neurodegeneration. Additionally, our findings introduce titin as a previously unrecognized component of macrophage biology and identify a mechanobiologically specialized macrophage program at the borders of the human brain. By demonstrating that TTN^+^ macrophages display distinct transcriptional signatures, spatial organization, and disease-associated remodeling, this study expands the conceptual framework of immune mechanotransduction and implicates TTN in neuroimmune function in disease.

Our findings extend recent models of ChP macrophage heterogeneity and dysfunction. Du et al. identified three principal ChP macrophage populations: CD163^+^, MHCII^+^, and CD9^+^ subsets, with distinct developmental origins and spatial niches.^9^ Consistent with this framework, we resolve multiple macrophage states spanning tissue-resident and monocyte-like identities, but additionally identify a TTN^+^ macrophage population characterized by coordinated activation of cytoskeletal, phagocytic, and stress-response programs. Our results also align with prior work demonstrating that the ChP functions as a dynamic immune interface in systemic infection, with regulated leukocyte trafficking and cytokine signaling shaping CNS immunity.^56^ In the context of AD, our findings further extend observations in the APP/PS1 mouse model, in which early epithelial dysfunction, including impaired barrier integrity and altered secretion of macrophage-regulatory factors, was observed.^12^ In human AD, we similarly observe broad, cell type-specific transcriptional remodeling across epithelial, endothelial, and stromal compartments. However, our data further indicate that macrophages undergo a coordinated state transition in AD, including expansion of a TTN^+^ senescent population and reduced ligand-receptor connectivity with neighboring cells. These findings support a model in which ChP dysfunction in AD reflects coupled alterations in epithelial and immune compartments, linking barrier disruption with macrophage reprogramming and loss of tissue integration.

Future work should determine whether TTN directly regulates macrophage mechanics, migration, or adhesion in the ChP; whether specific TTN isoforms confer specialized biomechanical properties; and whether mechanobiology-associated pathways can be therapeutically modulated to modulate cell migration and crosstalk in AD. More broadly, these results raise the possibility that mechanical changes at CNS interfaces contribute to the immune dysregulation characteristic of neurodegenerative disease.

### Limitations of the study

This work is exploratory in scope. The donor cohort is modest in size and cross-sectional, so the AD-associated changes described here are correlative, and larger, severity-stratified cohorts will be needed to determine whether TTN^+^ macrophage abundance tracks with disease progression. Detection of TTN is also shaped by the assays used: single-nucleus sequencing under-recovers exceptionally long transcripts, which is why we paired sequencing with RT-qPCR, RNAscope, and immunofluorescence rather than relying on any single modality. Finally, the functional consequences of macrophage TTN expression, including whether it is required for the cytoskeletal and mechanosensory programs with which it is associated, remain to be tested directly in experimental systems.

## RESOURCE AVAILABILITY

### Lead contact

Further information and requests for resources and reagents should be directed to and will be fulfilled by the lead contact, Marcello DiStasio (marcello.distasio@yale.edu).

### Materials availability

This study did not generate new unique reagents.

### Data and code availability

- The snRNA-seq and spatial transcriptomics data generated in this study have been deposited in the NCBI Gene Expression Omnibus (GEO) under accession numbers GSE315551 and GSE315553, respectively. Processed count matrices, cell annotations, and metadata used for all analyses are included in the GEO record. Additional data supporting the findings of this study are available from the corresponding author upon reasonable request.
- Custom analysis code is available at https://github.com/distasiolab/SHiPBIO (initial release https://doi.org/10.5281/zenodo.21516630).

## STAR★METHODS

### EXPERIMENTAL MODEL AND STUDY PARTICIPANT DETAILS

Tissues were obtained from deidentified postmortem human donors to the Yale Legacy Tissue Donation Program; use of deidentified postmortem human tissue was reviewed and approved by the Yale Institutional Review Board under protocol #2000032009. All donors or next of kin were consented for participation. Donor metadata is provided in Supplemental Materials Table S1.

### METHOD DETAILS

#### ChP tissue preparation

ChP tissues were dissected from the bilateral lateral ventricles. ChP samples were divided, with portions of tissue >10 grams snap frozen and a portion fixed in 10% formalin and embedded in paraffin.

#### Single-nucleus RNA sequencing

Nuclei were isolated on ice using the Nuclei EZ Prep Kit (NUC-101, Sigma-Aldrich) according to the manufacturer’s instructions, with some modifications. All procedures were carried out on ice or at 4°C. Briefly, frozen tissue was subjected to dounce homogenization using the KIMBLE Dounce Tissue Grinder Set (Sigma) and EZ Lysis buffer. After lysis, the sample was centrifuged and supernatants were discarded. Isolated nuclei were further processed using EZ lysis buffer and ice-cold Nuclei Suspension Buffer (1× PBS containing 0.01% BSA and 0.1% RNAse inhibitor), before being resuspended in Nuclei EZ Storage buffer and passed through a 40 μm nylon cell strainer. The nuclei suspensions were counted with trypan blue using a hemocytometer under a microscope. RNA libraries of barcoded single nuclei were assembled in compliance with the 10× Chromium Next GEM Single Cell 3^′^ platforms manufacturer procedures. Sequencing was performed by the Yale Center for Genome Analysis (YCGA) using an Illumina NovaSeq 6000, ensuring high-quality single nucleus RNA sequencing data for downstream analysis. The raw data obtained from sequencing was converted to fastq files, which were then aligned to the human reference genome (GRCh38) using the 10× Cell Ranger 7.1.0 pipeline.

#### Slide-seq spatial transcriptomics

OCT-embedded snap frozen tissues were cryosectioned at 10 μm thickness and applied to barcoded Curio Bioscience Seeker v1.1 3 × 3mm slide-seq tiles according to the manufacturer’s protocol. Orientations were not standardized because of free-hand embedding plane, chosen to preserve the folded epithelial–stromal architecture of the tissue. Near-consecutive sections were used for spatial transcriptomics and matched histologic assessment where applicable.

Sections were fixed, permeabilized, and processed for spatially resolved cDNA synthesis using Slide-seq reagents. Libraries were prepared and sequenced using an Illumina NovaSeq 6000, achieving a mean spatial resolution of 10 μm per bead. Raw sequencing data were processed using the Curio computational pipeline implemented in Nextflow.

#### CD163^+^ cell selection, RNA extraction, and cDNA synthesis

RNA was extracted from CD163 microbead-separated macrophages (Miltenyi Biotec, Cat. #130-124-420) using the RNeasy Mini Kit (Qiagen), following the manufacturer’s protocol. The extracted RNA was quantified, and its purity and integrity were assessed using a NanoDrop 2000 spectrophotometer (Thermo Scientific). For cDNA synthesis, 12 ng of RNA-equivalent cDNA was generated. The cDNA synthesis was performed using the iScript cDNA Synthesis Kit (Bio-Rad, Cat. #1708891), ensuring high-quality template for subsequent qPCR analysis. The cDNA was subsequently amplified using TTN-specific primers (IDT DNA; For sequences of primers used, see key resources table.

#### Validation and analysis of cDNA using qPCR

The TTN expression levels were assessed using quantitative PCR (qPCR) conducted with a Bio-Rad system. The generated cDNA was validated using qPCR, and the amplification plots were analyzed with Bio-Rad CFX Maestro software. This analysis provided detailed amplification profiles of the expression of TTN in the CD163 microbead-separated macrophages, with GAPDH serving as a control.

Relative TTN isoform expression was calculated by the ΔΔ*Cq* method: for each primer set, *Cq* values were first normalized to GAPDH within each sample (Δ*Cq* = *Cq*_*TTN*_ *Cq_GAPDH_*), and the Δ*Cq* of CD163 bead-enriched macrophages was then referenced to that of the paired unenriched whole ChP tissue (ΔΔ*Cq* = Δ*Cq_enriched_* Δ*Cq_whole_*), with fold change in enriched relative to whole tissue reported as 2^−ΔΔ*Cq*^.

#### Immunofluorescence and RNA *in situ* hybridization

Formalin-fixed, paraffin-embedded (FFPE) tissue blocks were sectioned into 5 μm thick slices and mounted on Superfrost Plus microscope slides. The slides were baked overnight at 40°C to ensure adherence of the sections, deparaffinized, and hydrated prior to staining.

For titin immunofluorescence, primary antibody incubation with rabbit anti-titin (Proteintech Cat. #27867-1-AP) (1:250), guinea pig anti-Pan-cytokeratin antibody (Origene Cat. #BP5069) (1:75), sheep anti-vWF (Invitrogen Cat. #PA1-43057) (1:500), and chicken anti-Iba1 antibody (Synaptic Systems Cat. #234 009) (1:500) was conducted overnight at 4°C, followed by detection with secondary donkey anti-rabbit Alexa Fluor 647 (Invitrogen, Cat. #A31573) (1:500), goat anti-guinea pig Alexa Fluor 488 (Invitrogen Cat. #A11073) (1:500), donkey anti-sheep Alexa Fluor 555 (Invitrogen Cat. #A21436) (1:500), and goat anti-chicken Alexa Fluor 594 (Invitrogen, Cat. #A11042) (1:500). Nuclei were counterstained with DAPI.

RNA probes were obtained from ACDBio, Inc., Newark, CA, for TTN (RNAscope Probe Hs-TTN, Cat. #550361) and detected using TSA Vivid Fluorophore Kit 650 (Tocris Bioscience, Cat. #7527). RNAscope was performed following manufacturer’s protocol. Micro-graphs for positive and negative control probes are shown in Figure S4. Primary antibody incubation with guinea pig anti-pan Cytokeratin antibody (Origene Cat. #BP5069) (1:75), sheep anti-vWF (Invitrogen Cat. #PA1-43057) (1:500), and chicken anti-Iba1 antibody (Synaptic Systems Cat. #234 009) (1:500) was conducted overnight at 4°C, followed by detection with secondary goat anti-guinea pig Alexa Fluor 488 (Invitrogen Cat. #A11073) (1:500), donkey anti-sheep Alexa Fluor 555 (Invitrogen Cat. #A21436) (1:500), and goat anti-chicken Alexa Fluor 594 (Invitrogen, Cat. #A11042) (1:500). Nuclei were counterstained with DAPI.

Slides were imaged on an inverted fluorescence microscope (Thunder Imager, Leica Microsystems, Wetzlar, Germany). For RNAscope quantification, only Iba1 immunofluorescence was used (for example images see Figure S3). Multiple slides imaged per condition, with a mean of 27.2 images per slide and a mean of 34.6 images per case. The total number of macrophages and the number of macrophages with probe signal in areas of 558.14 μm × 348.84 μm were recorded and pooled based on condition (AD or control). A total of 312 images were analyzed: 91 images from control slides (*n* = 2) and 221 images from AD slides (*n* = 7). Statistical analyses were performed using a Bayesian independent sample *t* test (H_*a*_ = AD > Control).

#### DAB immunohistochemistry

FFPE sections were deparaffinized in xylene and rehydrated using graded alcohols. Antigen retrieval was performed using citrate buffer, followed by blocking with 1% BSA and 0.3% Triton X-100. Primary antibody incubation with rabbit anti-CD163 (Abcam Inc., Cat. #ab182422) (1:200) was conducted overnight at 4°C. Detection was with biotinylated goat anti-rabbit IgG and ABC reagent (Vector Laboratories Cat. #PK-6101), with visualization via DAB substrate.

### QUANTIFICATION AND STATISTICAL ANALYSIS

#### Computational analysis

The snRNA-seq data was analyzed using Python-based tools, including scanpy v1.11.3^21^ scvi-tools v1.3.2^20^, and associated packages. Additional custom Python code was used for spatial ROI selection and analysis (see Data Availability section).

Quality control filtering was performed in Python using scanpy. Nuclei with low-complexity profiles or high mitochondrial transcript content were excluded on a sample-by-sample basis based on visual inspection of distributions followed by upper and lower thresholds set in multiples of median absolute deviations of the following criteria: total detected genes, total UMIs, and % mitochondrial reads. Genes detected in fewer than 2 nuclei were removed. Ambient RNA correction was not applied. Because these data were generated from isolated nuclei, ambient cytoplasmic RNA contamination was expected to be less prominent than in whole-cell datasets. We therefore prioritized standard quality-control filtering and downstream validation, ensuring that cell classes were clearly separable on the basis of canonical marker genes. After quality control, data from individual donors were integrated using scVI for batch correction and latent-space modeling.

To integrate single-nucleus RNA sequencing data across donors and conditions, we used a variational autoencoder framework implemented in scvi-tools. Raw count data were stored in the counts layer of an anndata object and used as input to the model. The anndata object was configured using SCVI.setup_anndata with donor identity (sample) specified as a batch covariate and disease condition (condition) included as an observed label. An SCVI model was trained with default parameters and a fixed random seed (seed = 0) to ensure reproducibility. The trained model was used to compute a low-dimensional latent representation of each nucleus, capturing biological variation while correcting for batch effects across samples. In addition, normalized gene expression values were obtained from the model using a library size of 10,000 counts per cell.

For downstream visualization and clustering, principal component analysis (PCA) was applied to the scVI latent space, followed by UMAP embedding. A semi-supervised UMAP was generated by incorporating cell class labels as guidance during embedding. Nearest-neighbor graph construction and Leiden clustering were performed on the scVI-derived representation, and clusters were annotated based on the most frequent marker-based cell class assignment within each cluster.

#### Differential gene expression

To define transcriptional changes between conditions and macrophage subpopulations, we performed differential gene expression (DGE) analysis on scVI-corrected expression values using scanpy.tl.rank_genes_groups with the Wilcoxon rank-sum test, and genes were ranked by adjusted *p*-value and fold change. Where applicable, adjusted *p*-values were corrected for multiple testing using the Benjamini-Hochberg procedure. For visualization, log_2_ fold changes are reported. To focus on robust and biologically meaningful differences, we applied a threshold of log_2_FC > 0.5 and adjusted *p*-value <0.05.

#### Cell composition analysis

To characterize the baseline cellular architecture of the tissue independent of experimental perturbation, we examined the intercept terms of a scCODA model fitted to our data, which represent the log-scale baseline abundances of each cell type relative to the reference population. scCODA implements a Bayesian multinomial–logit framework with a spike-and-slab prior, allowing simultaneous estimation of positive, negative, or null compositional effects while accounting for the inherent sum-to-one constraints of compositional data. By transforming the posterior mean intercepts through a softmax function, we derived an interpretable baseline composition profile reflecting the expected proportion of each cell type before conditioning on the experimental variable. This intercept-derived composition provides a model-based estimate of the underlying cellular milieu, integrating information across all samples while adjusting for reference constraints and compositional dependencies. In our dataset, the resulting stacked bar representation highlights the baseline dominance of specific populations and provides a principled point of comparison for evaluating subsequent condition-associated shifts in relative abundance.

#### Gene module scoring

Gene module scores were computed using scanpy.tl.score_genes, which measures the average expression of a predefined gene set in each cell relative to a background reference set of matched genes. Scores are centered around zero, such that positive values indicate enrichment of the module and negative values indicate depletion relative to background. Gene sets for scoring were curated from a combination of publicly available pathway databases (MSigDB, Reactome), ImmGen and human myeloid single-cell atlases, and manual literature curation. Specific genes used for myeloid cell types (Figure 1F) are listed in Table S3, and genes used for functional annotations (Figure 3A) are listed in Table S5. Unless otherwise specified, statistical tests on proportions were performed at the donor level.

#### Gene set enrichment analysis and senescence scoring

To investigate coordinated pathway-level changes, we performed gene set enrichment analysis (GSEA) using Enrichr^57^ across Gene Ontology Biological Process, KEGG, and Reactome databases. Enrichment statistics were derived from the ranked DGE results. To quantitatively assess cellular aging programs, we used SenePy,^58^ a framework for single-cell resolution senescence scoring. SenePy computes enrichment scores for curated senescence gene sets while adjusting for dataset-specific expression variability.

#### Cell interaction analysis

Cell-cell communication was inferred using LIANA,^28^ which provides consensus scoring across multiple ligand-receptor inference algorithms to ensure robustness to model-specific biases. We applied LIANA to scVI-normalized expression profiles in our snRNA-seq data to quantify the probability of active ligand-receptor interactions between all annotated cell classes. Ligand-receptor interactions were filtered based on statistical significance (*p* < 0.05) and ranked by scaled weight, with the top interactions retained for visualization. Consensus scores were aggregated and visualized using circos plots to highlight directional communication patterns in control and Alzheimer’s disease tissue.

#### Atlas integration

To contextualize ChP macrophages within a broader myeloid landscape, we performed cross-dataset integration between our macrophage snRNA-seq data and the myeloid compartment of a cross tissue immune cell atlas^24^ using a non-negative matrix factorization (NMF) framework implemented in python with the scikit-learn library. NMF decomposes high-dimensional single-nucleus gene expression matrices into a set of biologically interpretable latent factors whose non-negativity constraints promote sparse, additive representations of cellular programs. For integration, we projected both our ChP dataset and external reference atlases of human myeloid populations into a shared NMF-derived factor space. This approach aligns gene expression profiles across datasets without imposing hard cluster boundaries and enables robust comparison of transcriptional identities across tissues. The resulting joint factorization allowed us to map ChP macrophages onto well-defined myeloid states.

#### Spatial transcriptomic data analysis

Spatial barcode deconvolution, gene count matrices, and spatial alignment were performed using the Curio pipeline and validated with gene signatures and anatomical landmarks. After batch correction with Harmony,^59^ we used GIMVI^27^ to integrate our ChP-derived snRNA-seq dataset into the spatial transcriptome dataset to increase gene coverage. We then used CellCharter^60^ to perform graph-based coarse neighborhood clustering, and identified cell types using gene signature scores for canonical cell-type marker sets, assigning each cell to the class whose marker signature achieved the maximal score for that cell.

#### Spatial neighborhood analysis

To integrate transcriptional states with spatial organization in the ChP, we performed a multimodal spatial neighborhood analysis combining cross-modal imputation, graph-based clustering, and neighborhood enrichment statistics. First, we used GIMVI,^27^ a variational inference framework that jointly models snRNA-seq and spatial transcriptomics data, to impute high-resolution gene expression profiles into spatial RNA expression profiles. This approach enables transfer of macrophage subcluster signatures onto the tissue architecture while preserving spatial covariance across modalities. We then applied CellCharter,^60^ which performs graph-based clustering on spatial expression graphs, to delineate coherent anatomical and functional domains within the ChP. These domains captured epithelial folds, stromal regions, and immune-enriched microenvironments and provided a spatial scaffold for downstream analyses. Squidpy’s neighborhood enrichment framework^61^ was used to quantify the overrepresentation of specific cell types in the immediate proximity of TTN-positive and TTN-negative macrophages. Neighborhood enrichment around macrophage subclusters was quantified as the log_2_ ratio of neighborhood frequency to global frequency. Statistical significance was assessed with a permutation-based test in which non-macrophage cell labels were randomly shuffled within each sample while preserving spatial coordinates and macrophage center identities, thereby maintaining sample-specific composition and spatial structure. For each macrophage subtype and neighboring cell type, empirical *p*-values were calculated from 2,000 permutations based on the null distribution of log_2_ enrichment. *p*-values were then combined across samples using Fisher’s method to obtain a single significance estimate per cell type-macrophage subtype pair.

## Supplementary Material

1

Supplementary data related to this article can be found online at https://doi.org/10.1016/j.celrep.2026.117859.

## Figures and Tables

**Table:** KEY RESOURCES TABLE

REAGENT or RESOURCE	SOURCE	IDENTIFIER
Antibodies		
anti-titin	Proteintech	Cat# 27867-1-AP; RRID: AB_2880998
anti-Pan-cytokeratin	Origene	Cat# BP5069; RRID: AB_979529
anti-vWF	Invitrogen	Cat # PA1-43057; RRID: AB_1088286
anti-Iba1	Synaptic Systems	Cat#: 234 009; RRID: AB_2891282
anti-CD163	Abcam Inc	Cat#: ab182422; RRID: AB_2753196
Critical commercial assays		
Slide-seq chip	Curio Bioscience	Seeker v1.1 3 × 3mm
Single Nucleus Sequencing chip	10× Genomics	Chromium Next GEM Single Cell 3’ v3.1
Deposited data		
Human ChP snRNA-seq data	GEO	GSE315551
Human ChP spatial transcriptomics data	GEO	GSE315553
Oligonucleotides		
PCR primer set 1	Integrated DNA Technologies Coralville, IA	5′-ATGAGCAATGGAGACCTAACAG-3’ (forward)
		5′-CCGAAGCCAAAGTCAAGATCC-3’ (reverse)
PCR primer set 3	Integrated DNA Technologies Coralville, IA	5′-CAGGTTCCTTCTTAGGCACAG-3’ (forward)
		5′-GAGGTGTCAGTCACAGTCCC-3’ (reverse)
PCR primer set 7	Integrated DNA Technologies Coralville, IA	5′-GCAGCCTCTTCCTTAGGTGC-3’ (forward)
		5′-CAAACCACCTCCTGTGGAACC-3’ (reverse)
PCR primer set 12	Integrated DNA Technologies Coralville, IA	5′-CTGCTGCGACACTCTATGAC-3’ (forward)
		5′-CTAAATTAACCATCCGTGAAACC-3’ (reverse)
Software and algorithms		
SHiPBio	Github	https://doi.org/10.5281/zenodo.21516630

## Highlights

- snRNA-seq of human choroid plexus resolves border-associated macrophage diversity
- A TTN-expressing macrophage population occupies the choroid plexus
- TTN^+^ macrophages are expanded in choroid plexus of donors with Alzheimer’s disease
- Multiplex imaging confirms Iba1^+^ TTN^+^ cells in perivascular choroid plexus stroma

## References

[1] Xu H, Lotfy P, Gelb S, Pragana A, Hehnly C, Byer LI, Shipley FB, Zawadzki ME, Cui J, Deng L, (2024). The choroid plexus synergizes with immune cells during neuroinflammation. Cell 187, 4946–4963.e17. 10.1016/j.cell.2024.07.002. https://www.cell.com/cell/abstract/S0092-8674(24)00717-7. 39089253 PMC11458255

[2] Van Hove H, Glück C, Mildenberger W, Petrova E, Maheshwari U, Häne P, Kreiner V, Bijnen M, Mussak C, Utz SG, (2025). Inter-leukin-34-dependent perivascular macrophages promote vascular function in the brain. Immunity 58, 1289–1305.e8. 10.1016/j.immuni.2025.04.003. https://www.sciencedirect.com/science/article/pii/S1074761325001669. 40315842

[3] Utz SG, See P, Mildenberger W, Thion MS, Silvin A, Lutz M, Ingelfinger F, Rayan NA, Lelios I, Buttgereit A, (2020). Early Fate Defines Microglia and Non-parenchymal Brain Macrophage Development. Cell 181, 557–573.e18. 10.1016/j.cell.2020.03.021. https://www.cell.com/cell/abstract/S0092-8674(20)30283-X. 32259484

[4] Guilliams M, Thierry GR, Bonnardel J, and Bajenoff M. (2020). Establishment and Maintenance of the Macrophage Niche. Immunity 52, 434–451. 10.1016/j.immuni.2020.02.015. 32187515

[5] Goldmann T, Wieghofer P, Jordão MJC, Prutek F, Hagemeyer N, Frenzel K, Amann L, Staszewski O, Kierdorf K, Krueger M, (2016). Origin, fate and dynamics of macrophages at central nervous system interfaces. Nat. Immunol. 17, 797–805. 10.1038/ni.3423. 27135602 PMC4968048

[6] Mrdjen D, Pavlovic A, Hartmann FJ, Schreiner B, Utz SG, Leung BP, Lelios I, Heppner FL, Kipnis J, Merkler D, (2018). High-Dimensional Single-Cell Mapping of Central Nervous System Immune Cells Reveals Distinct Myeloid Subsets in Health, Aging, and Disease. Immunity 48, 380–395.e6. 10.1016/j.immuni.2018.01.011. 29426702

[7] Sankowski R, Süß P, Benkendorff A, Böttcher C, Fernandez-Zapata C, Chhatbar C, Cahueau J, Monaco G, Gasull AD, Khavaran A, (2024). Multiomic spatial landscape of innate immune cells at human central nervous system borders. Nat. Med. 30, 186–198. 10.1038/s41591-023-02673-1. 38123840 PMC10803260

[8] Van Hove H, Martens L, Scheyltjens I, De Vlaminck K, Pombo An-tunes AR, De Prijck S, Vandamme N, De Schepper S, Van Isterdael G, Scott CL, (2019). A single-cell atlas of mouse brain macrophages reveals unique transcriptional identities shaped by ontogeny and tissue environment. Nat. Neurosci. 22, 1021–1035. 10.1038/s41593-019-0393-4. 31061494

[9] Du S, Nguyen KM, Ulezko Antonova A, Fachi JL, Rodrigues PF, Verdiani A, Molgora M, Smirnov I, Herz J, Mamuladze T, (2026). Diversity and immune dynamics of choroid plexus macrophages are shaped by distinct developmental origins. Nat. Neurosci. 29, 717–731. 10.1038/s41593-025-02158-z. 41566006 PMC13178392

[10] Vara-Pérez M, and Movahedi K. (2025). Border-associated macrophages as gatekeepers of brain homeostasis and immunity. Immunity 58, 1085–1100. 10.1016/j.immuni.2025.04.005. 40324381 PMC12094687

[11] Čarna M, Onyango IG, Katina S, Holub D, Novotny JS, Nezvedova M, Jha D, Nedelska Z, Lacovich V, Vyvere TV, (2023). Pathogenesis of Alzheimer’s disease: Involvement of the choroid plexus. Alzheimer’s Dement. 19, 3537–3554. 10.1002/alz.12970. 36825691 PMC10634590

[12] Yan Z-J, Ye M, Li J, Zhang DF, and Yao YG. (2025). Early transcriptional and cellular abnormalities in choroid plexus of a mouse model of Alzheimer’s disease. Mol. Neurodegener. 20, 62. 10.1186/s13024-025-00853-w. 40450296 PMC12125878

[13] Strominger I, Elyahu Y, Berner O, Reckhow J, Mittal K, Nemirovsky A, and Monsonego A. (2018). The Choroid Plexus Functions as a Niche for T-Cell Stimulation Within the Central Nervous System. Front. Immunol. 9, 1066–3224. 10.3389/fimmu.2018.01066. 29868025 PMC5962702

[14] Baruch K, Deczkowska A, David E, Castellano JM, Miller O, Kertser A, Berkutzki T, Barnett-Itzhaki Z, Bezalel D, Wyss-Coray T, (2014). Aging-induced type I interferon response at the choroid plexus negatively affects brain function. Science 346, 89–93. 10.1126/science.1252945. 25147279 PMC4869326

[15] Du H, Bartleson JM, Butenko S, Alonso V, Liu WF, Winer DA, and Butte MJ. (2023). Tuning immunity through tissue mechanotransduction. Nat. Rev. Immunol. 23, 174–188. 10.1038/s41577-022-00761-w. 35974148 PMC9379893

[16] Oakes PW, Wagner E, Brand CA, Probst D, Linke M, Schwarz US, Glotzer M, and Gardel ML. (2017). Optogenetic control of RhoA reveals zyxin-mediated elasticity of stress fibres. Nat. Commun. 8, 15817. 10.1038/ncomms15817. 28604737 PMC5477492

[17] Dustin ML, and Cooper JA. (2000). The immunological synapse and the actin cytoskeleton: molecular hardware for T cell signaling. Nat. Immunol. 1, 23–29. 10.1038/76877. 10881170

[18] McWhorter FY, Wang T, Nguyen P, Chung T, and Liu WF. (2013). Modulation of macrophage phenotype by cell shape. Proc. Natl. Acad. Sci. USA 110, 17253–17258. 10.1073/pnas.1308887110. 24101477 PMC3808615

[19] Zhang X, Kim T-H, Thauland TJ, Li H, Majedi FS, Ly C, Gu Z, Butte MJ, Rowat AC, and Li S. (2020). Unraveling the mechanobiology of immune cells. Curr. Opin. Biotechnol. 66, 236–245. 10.1016/j.copbio.2020.09.004. 33007634 PMC7524653

[20] Lopez R, Regier J, Cole MB, Jordan MI, and Yosef N. (2018). Deep generative modeling for single-cell transcriptomics. Nat. Methods 15, 1053–1058. 10.1038/s41592-018-0229-2. 30504886 PMC6289068

[21] Wolf FA, Angerer P, and Theis FJ. (2018). SCANPY: large-scale single-cell gene expression data analysis. Genome Biol. 19, 15. 10.1186/s13059-017-1382-0. 29409532 PMC5802054

[22] Büttner M, Ostner J, Müller CL, Theis FJ, and Schubert B. (2021). scCODA is a Bayesian model for compositional single-cell data analysis. Nat. Commun. 12, 6876. 10.1038/s41467-021-27150-6. 34824236 PMC8616929

[23] Tirosh I, Izar B, Prakadan SM, Wadsworth MH, Treacy D, Trombetta JJ, Rotem A, Rodman C, Lian C, Murphy G, (2016). Dissecting the multicellular ecosystem of metastatic melanoma by single-cell RNA-seq. Science 352, 189–196. 10.1126/science.aad0501. 27124452 PMC4944528

[24] Domínguez Conde C, Xu C, Jarvis LB, Rainbow DB, Wells SB, Gomes T, Howlett SK, Suchanek O, Polanski K, King HW, (2022). Cross-tissue immune cell analysis reveals tissue-specific features in humans. Science 376, eabl5197. 10.1126/science.abl5197. 35549406 PMC7612735

[25] Dani N, Herbst RH, McCabe C, Green GS, Kaiser K, Head JP, Cui J, Shipley FB, Jang A, and Dionne D. (2021). A cellular and spatial map of the choroid plexus across brain ventricles and ages. Cell 184, 3056–3074.e21. 10.1016/j.cell.2021.04.003. 33932339 PMC8214809

[26] Rodriques SG, Stickels RR, Goeva A, Martin CA, Murray E, Vanderburg CR, Welch J, Chen LM, Chen F, and Macosko EZ. (2019). Slide-seq: A scalable technology for measuring genome-wide expression at high spatial resolution. Science 363, 1463–1467. 10.1126/science.aaw1219. 30923225 PMC6927209

[27] Lopez R, Nazaret A, Langevin M, Samaran J, Regier J, Jordan MI, and Yosef N. (2019). A joint model of unpaired data from scRNA-seq and spatial transcriptomics for imputing missing gene expression measurements. Preprint at ArXiv. 10.48550/arXiv.1905.02269.

[28] Dimitrov D, Schäfer PSL, Farr E, Rodriguez-Mier P, Lobentanzer S, Badia-i-Mompel P, Dugourd A, Tanevski J, Ramirez Flores RO, and Saez-Rodriguez J. (2024). LIANA+ provides an all-in-one framework for cell–cell communication inference. Nat. Cell Biol. 26, 1613–1622. 10.1038/s41556-024-01469-w. 39223377 PMC11392821

[29] Zeisel A, Muñoz-Manchado AB, Codeluppi S, Lönnerberg P, La Manno G, Juréus A, Marques S, Munguba H, He L, Betsholtz C, (2015). Cell types in the mouse cortex and hippocampus revealed by single-cell RNA-seq. Science 347, 1138–1142. 10.1126/science.aaa1934. 25700174

[30] Shechter R, London A, Varol C, Raposo C, Cusimano M, Yovel G, Rolls A, Mack M, Pluchino S, Martino G, (2009). Infiltrating Blood-Derived Macrophages Are Vital Cells Playing an Anti-inflammatory Role in Recovery from Spinal Cord Injury in Mice. PLoS Med. 6, e1000113. 10.1371/journal.pmed.1000113. 19636355 PMC2707628

[31] London A, Itskovich E, Benhar I, Kalchenko V, Mack M, Jung S, and Schwartz M. (2011). Neuroprotection and progenitor cell renewal in the injured adult murine retina requires healing monocyte-derived macrophages. J. Exp. Med. 208, 23–39. 10.1084/jem.20101202. 21220455 PMC3023128

[32] Toffali L, D’Ulivo B, Giagulli C, Montresor A, Zenaro E, Delledonne M, Rossato M, Iadarola B, Sbarbati A, Bernardi P, (2023). An isoform of the giant protein titin is a master regulator of human T lymphocyte trafficking. Cell Rep. 42, 112516. 10.1016/j.celrep.2023.112516. 37204926

[33] de Andrade Silva BJ, Hughes TK, Andrade PR, Ma F, Teles RMB, Shalek AK, Bloom B, Pellegrini M, and Modlin RL. (2020). Single-cell RNA-sequencing of leprosy skin lesions reveals a titin (TTN) expressing T cell subset associated with the progressive disease. J. Immunol. 204, 225.36–236. 10.4049/jimmunol.204.Supp.225.36.

[34] Lӓmmermann T, and Germain RN. (2014). The multiple faces of leukocyte interstitial migration. Semin. Immunopathol. 36, 227–251. 10.1007/s00281-014-0418-8. 24573488 PMC4118216

[35] McWhorter FY, Davis CT, and Liu WF. (2014). Physical and mechanical regulation of macrophage phenotype and function. Cell. Mol. Life Sci. 72, 1303–1316. 10.1007/s00018-014-1796-8. 25504084 PMC4795453

[36] Mammoto A, Mammoto T, and Ingber DE. (2012). Mechanosensitive mechanisms in transcriptional regulation. J. Cell Sci. 125, 3061–3073. 10.1242/jcs.093005. 22797927 PMC3434847

[37] Zhao X, Di Q, Liu H, Quan J, Ling J, Zhao Z, Xiao Y, Wu H, Wu Z, Song W, (2022). MEF2C promotes M1 macrophage polarization and Th1 responses. Cell. Mol. Immunol. 19, 540–553. 10.1038/s41423-022-00841-w. 35194174 PMC8975968

[38] Deczkowska A, Matcovitch-Natan O, Tsitsou-Kampeli A, Ben-Hamo S, Dvir-Szternfeld R, Spinrad A, Singer O, David E, Winter DR, Smith LK, (2017). Mef2C restrains microglial inflammatory response and is lost in brain ageing in an IFN-I-dependent manner. Nat. Commun. 8, 717. 10.1038/s41467-017-00769-0. 28959042 PMC5620041

[39] Kierdorf K, Masuda T, Jordão MJC, and Prinz M. (2019). Macrophages at CNS interfaces: ontogeny and function in health and disease. Nat. Rev. Neurosci. 20, 547–562. 10.1038/s41583-019-0201-x. 31358892

[40] Bianco P, Nagy A, Kengyel A, Szatmári D, Mártonfalvi Z, Huber T, and Kellermayer MSZ. (2007). Interaction Forces between F-Actin and Titin PEVK Domain Measured with Optical Tweezers. Biophys. J. 93, 2102–2109. 10.1529/biophysj.107.106153. 17513381 PMC1959548

[41] Loescher CM, Hobbach AJ, and Linke WA. (2022). Titin (TTN): from molecule to modifications, mechanics, and medical significance. Cardiovasc. Res. 118, 2903–2918. 10.1093/cvr/cvab328. 34662387 PMC9648829

[42] Allen WE, Jones GE, Pollard JW, and Ridley AJ. (1997). Rho, Rac and Cdc42 regulate actin organization and cell adhesion in macrophages. J. Cell Sci. 110, 707–720. 10.1242/jcs.110.6.707. 9099945

[43] Aflaki E, Balenga NAB, Luschnig-Schratl P, Wolinski H, Povoden S, Chandak PG, Bogner-Strauss JG, Eder S, Konya V, Kohlwein SD, (2011). Impaired Rho GTPase activation abrogates cell polarization and migration in macrophages with defective lipolysis. Cell. Mol. Life Sci. 68, 3933–3947. 10.1007/s00018-011-0688-4. 21533980 PMC3214256

[44] Stramer B, Wood W, Galko MJ, Redd MJ, Jacinto A, Parkhurst SM, and Martin P. (2005). Live imaging of wound inflammation in Drosophila embryos reveals key roles for small GTPases during in vivo cell migration. J. Cell Biol. 168, 567–573. 10.1083/jcb.200405120. 15699212 PMC2171743

[45] Königs V, Jennings R, Vogl T, Horsthemke M, Bachg AC, Xu Y, Grobe K, Brakebusch C, Schwab A, Bähler M, (2014). Mouse macrophages completely lacking Rho subfamily GTPases (RhoA, RhoB, and RhoC) have severe lamellipodial retraction defects, but robust chemo-tactic navigation and altered motility. J. Biol. Chem. 289, 30772–30784. 10.1074/jbc.M114.563270. 25213860 PMC4215254

[46] Ridley AJ. (2001). Rho GTPases and cell migration. J. Cell Sci. 114, 2713–2722. 10.1242/jcs.114.15.2713. 11683406

[47] Meli VS, Veerasubramanian PK, Atcha H, Reitz Z, Downing TL, and Liu WF. (2019). Biophysical regulation of macrophages in health and disease. J. Leukoc. Biol. 106, 283–299. 10.1002/JLB.MR0318-126R. 30861205 PMC7001617

[48] Heneka MT, van der Flier WM, Jessen F, Hoozemanns J, Thal DR, Boche D, Brosseron F, Teunissen C, Zetterberg H, Jacobs AH, (2025). Neuroinflammation in Alzheimer disease. Nat. Rev. Immunol. 25, 321–352. 10.1038/s41577-024-01104-7. 39653749 PMC13148177

[49] Ito M, Komai K, Mise-Omata S, Iizuka-Koga M, Noguchi Y, Kondo T, Sakai R, Matsuo K, Nakayama T, Yoshie O, (2019). Brain regulatory T cells suppress astrogliosis and potentiate neurological recovery. Nature 565, 246–250. 10.1038/s41586-018-0824-5. 30602786

[50] Zhang Y, Fung ITH, Sankar P, Chen X, Robison LS, Ye L, D’Souza SS, Salinero AE, Kuentzel ML, Chittur SV, (2020). Depletion of nk cells improves cognitive function in the alzheimer disease mouse model. J. Immunol. 205, 502–510. 10.4049/jimmunol.2000037. 32503894 PMC7343613

[51] Zenaro E, Pietronigro E, Bianca VD, Piacentino G, Marongiu L, Budui S, Turano E, Rossi B, Angiari S, Dusi S, (2015). Neutrophils promote Alzheimer’s disease–like pathology and cognitive decline via LFA-1 integrin. Nat. Med. 21, 880–886. 10.1038/nm.3913. 26214837

[52] Hong S, Beja-Glasser VF, Nfonoyim BM, Frouin A, Li S, Ramakrishnan S, Merry KM, Shi Q, Rosenthal A, Barres BA, (2016). Complement and microglia mediate early synapse loss in Alzheimer mouse models. Science 352, 712–716. 10.1126/science.aad8373. 27033548 PMC5094372

[53] Hansen DV, Hanson JE, and Sheng M. (2018). Microglia in Alzheimer’s disease. JCB (J. Cell Biol.) 217, 459–472. 10.1083/jcb.201709069. 29196460 PMC5800817

[54] Shechter R, Miller O, Yovel G, Rosenzweig N, London A, Ruckh J, Kim KW, Klein E, Kalchenko V, Bendel P, (2013). Recruitment of beneficial M2 macrophages to injured spinal cord is orchestrated by remote brain choroid plexus. Immunity 38, 555–569. 10.1016/j.immuni.2013.02.012. 23477737 PMC4115271

[55] Shechter R, London A, and Schwartz M. (2013). Orchestrated leukocyte recruitment to immune-privileged sites: absolute barriers versus educational gates. Nat. Rev. Immunol. 13, 206–218. 10.1038/nri3391. 23435332

[56] Fullard JF, Lee H-C, Voloudakis G, Suo S, Javidfar B, Shao Z, Peter C, Zhang W, Jiang S, Corvelo A, (2021). Single-nucleus transcriptome analysis of human brain immune response in patients with severe COVID-19. Genome Med. 13, 118. 10.1186/s13073-021-00933-8. 34281603 PMC8287557

[57] Chen EY, Tan CM, Kou Y, Duan Q, Wang Z, Meirelles GV, Clark NR, and Ma’ayan A. (2013). Enrichr: interactive and collaborative HTML5 gene list enrichment analysis tool. BMC Bioinf. 14, 128. 10.1186/1471-2105-14-128. PMC3637064 23586463

[58] Sanborn MA, Wang X, Gao S, Dai Y, and Rehman J. (2025). Unveiling the cell-type-specific landscape of cellular senescence through single-cell transcriptomics using SenePy. Nat. Commun. 16, 1884. 10.1038/s41467-025-57047-7. 39987255 PMC11846890

[59] Korsunsky I, Millard N, Fan J, Slowikowski K, Zhang F, Wei K, Baglaenko Y, Brenner M, Loh PR, and Raychaudhuri S. (2019). Fast, sensitive and accurate integration of single-cell data with Harmony. Nat. Methods 16, 1289–1296. 10.1038/s41592-019-0619-0. 31740819 PMC6884693

[60] Varrone M, Tavernari D, Santamaria-Martínez A, Walsh LA, and Ciriello G. (2024). CellCharter reveals spatial cell niches associated with tissue remodeling and cell plasticity. Nat. Genet. 56, 74–84. 10.1038/s41588-023-01588-4. 38066188

[61] Palla G, Spitzer H, Klein M, Fischer D, Schaar AC, Kuemmerle LB, Rybakov S, Ibarra IL, Holmberg O, Virshup I, (2022). Squidpy: a scalable framework for spatial omics analysis. Nat. Methods 19, 171–178. 10.1038/s41592-021-01358-2. 35102346 PMC8828470

