# Supplementary material for: TTN+ macrophages are enriched in the human choroid plexus in Alzheimer’s disease
Source: Cell Rep. Author manuscript; Available in PMC 2026 Sep 15. (PMC13575895; doi:10.1016/j.celrep.2026.117859)
Supplement: Supplementary file 1 [file NIHMS2206933-supplement-1.pdf]

Cell Reports, Volume *45*

Supplemental information

TTN$^+$ macrophages are enriched in the human choroid plexus in Alzheimer's disease

Sagar Bhatta, Alayna Grzybowski, Gion Ortiz, and Marcello DiStasio

1 Supplemental Tables

Subject	Age	Sex	PMI	Dementia	Pathologic/Clinical History	snRNA seq	RNA scope	Spatial RNAseq	qPCR
A.1	36	M	11h	(-)	Cerebrovascular disease	✓			
A.2	66	F	25h	(-)	Squamous cell carcinoma of lung	✓			
A.3	78	M	23h	(-)	Pneumonia	✓			
A.4	68	F	32h	(-)	Myocardial infarction	✓			
A.5	82	M	22h	AD	High level of ADNC (A3,B3,C2)	✓		✓	
A.6	55	F	14h	AD	High level of ADNC (A3,B3,C3)	✓			
YA.1	69	F	12h	AD	High level of ADNC (A3,B3,C3)	✓			
YA.2	88	F	9h	AD	High level of ADNC (A3,B3,C3)	✓			
YA.3	79	M	5h	AD	Alzheimer's disease; pathologic stage unknown	✓			
YA.4	89	M	6h	AD	Intermediate level of ADNC (A1,B3,C2); Pneumonia			✓	
YA.5	87	F	4h	AD	Intermediate level of ADNC (A3,B2,C2)			✓	
YA.6	63	M	5h	AD	High level of ADNC (A3,B3,C2); LBD, transitional/limbic			✓	
A.7	77	M	114h	(-)	Pneumonia		✓		
A.8	70	M	38h	(-)	Pulmonary embolism		✓		
A.9	59	F	22h	AD	High level of ADNC (A3,B3,C3)		✓		
A.10	88	F	9h	AD	High level of ADNC (A3,B3,C3)		✓		
A.11	86	F	4h	AD	Alzheimer's disease; pathologic stage unknown		✓		
A.12	89	F	15h	AD	Intermediate level of ADNC (A2,B3,C2); Remote infarct, R. occipital cortex		✓		
A.13	84	F	41h	AD	Intermediate level of ADNC (A2,B3,C2)		✓		
A.14	94	F	45h	AD	Low level of ADNC (A1,B2,C2)		✓		
A.15	75	F	24h	(-)	Bronchopulmonary hemorrhage				ChP, FC, MesFat, SubFat, CSF
A.16	66	F	22h	(-)	Achondroplasia				ChP, FC, MesFat, SubFat
YA.7	62	F	4h	(-)	No history of cognitive disorder				ChP
YA.8	84	F	18h	AD	High level of ADNC (A3,B3,C3)				ChP
YA.9	88	M	6h	AD	High level of ADNC (A3,B3,C2)				ChP, FC, MesFat, SubFat
YA.10	73	M	4h	AD	AD neuropathologic stage unknown				ChP, FC
YA.11	39	M	4h	(-)	High grade glioma				ChP, FC
YA.12	55	F	5h	(-)	Catheter infection				ChP
YA.13	71	M	5h	(-)	Pneumonia				ChP

Table S1: Clinical and experimental metadata for donor samples used in this study. Abbreviations: **ChP**: choroid plexus; **AD**: Alzheimer's disease; **ADNC**: Alzheimer's disease neuropathologic change; LBD: Lewy body disease; **FC**: frontal cortex; **MesFat**: mesenteric fat; **SubFat**: subcutaneous fat; **CSF**: cerebrospinal fluid

Cell type	High-expression marker genes
Endothelial	FLT1, PECAM1
Glial	GFAP, SLC1A3, FABP7
Ependymal	FOXJ1, CIMAP3, DYNLRB2
Epithelial	TTR, KCNE2, KCNQ1, KCNA3, MUC1, EPCAM, KRT8, KRT18, AQP1, OTX2, CLDN1, CLDN2, CLDN3, SLC4A5, ATP1A1, PAX6, LAMP5, PLTP, GPR125
Macrophage	C1QA, C1QB, C1QC, CCR5, CD14, CD68, CD163, CD86, CLEC7A, CTSB, CX3CR1, DOCK2, DOCK4, FCGR1A, FCGR3A, ITGAX, MRC1, MSR1, PTPRC, TBXAS1, SLC8A1, TLR2
Neural	NEFL, MAPT, KCNQ5, RBFOX3, MAP2, TUBB3, DCX, NEFM, SYN1, SLC17A7, GAD1, GAD2, STMN2, SNAP25, CAMK2A, ELAVL4, GRIN1
Mesenchymal	NT5E, ENG, PDGFRA, COL1A1, COL1A2, DCN, LUM, ACTA2, TAGLN, RGS5, NOTCH3, MCAM, VIM, FN1, PDGFRB, THY1
T-cell	CD3D, CD3E, CD3G, THEMIS, CAMK4, CD52, IL32, LTB, IL7R, CD2, INPP4B, TRAC, TC2N, ADAM19, TRAT1, SPOCK2, ICOS, CXCR4, CD27, ITM2A, CD247
B-cell	MS4A1, CD19, EBF1, IGLV6-57, IGLV4-60, IGKV3D-20, IGKV6-21, IGKJ3, IGKJ4, KLHL14, CEP295NL, WNT16, GNG3, ICOSLG, OSBPL10, FCRL5
Dendritic cell	CD1A, CLEC4C, NRP1, CR2, FCER2, IL3RA, CD207, ITGAX, HLA-DRA, HLA-DRB1, HLA-DRB3

Table S2: Cell type gating markers used for annotation of choroid plexus cell populations

Module	Genes
Myeloid Identity	LYZ, CSF1R, SPI1, CD68, MPEG1
Granulocytic	MPO, ELANE, S100A8, S100A9, FCGR3B
Monocytic lineage	CD14, FCGR3A, CCR2, CX3CR1
Immature / progenitor	MKI67, TOP2A, LY6E, MPO
Circulating monocyte	S100A8, S100A9, CCR2, FCN1, VCAN
Tissue macrophage	MRC1, CD163, MSR1, MARCO, TREM2, APOE, FABP5, SPP1
Inflammatory	IL1B, TNF, NFKBIA, CXCL9, CXCL10, STAT1, IRF1, GBP1
Anti-inflammatory	MRC1, CD163, MSR1, C1QA, C1QB, C1QC, TREM2, APOE
Antigen presentation	HLA-DRA, HLA-DRB1, CD74, B2M, CIITA
Phagocytosis	MERTK, CD36, MARCO, MSR1, LPL, GPNMB
Brain microglia	TREM2, TMEM119, P2RY12, CX3CR1
Liver Kupffer	CLEC4F, VSIG4, CD5L, MARCO
Lung alveolar	SIGLEC1, MARCO, FABP4, PPARG, LPL
Retina Choroid	CD163, MRC1, TREM2, APOE, SPP1, LYVE1, MERTK

Table S3: Marker gene sets used to annotate myeloid lineage states, activation programs, and tissue-specialized macrophage populations.

Sample	Total Macs	TTN^+ Macs	Fraction TTN^+	Condition
A.1	168	29	0.173	control
A.2	82	12	0.146	control
A.3	20	2	0.100	control
A.4	79	16	0.203	control
YA.1	40	21	0.525	AD
YA.2	19	10	0.526	AD
YA.3	4	3	0.750	AD

Table S4: Donor-level proportions of TTN^+ macrophages in control and Alzheimer's disease (AD) choroid plexus samples.

Module	Genes
TTN regulatory factors	BACH1, FOS, JUN, JUNB, HSF1, MEF2A, MEF2C, MEF2D, IRF8, MAFB, TCF12, PKNOX1
Tissue Residence	CX3CR1, ITGAM, ITGAX, CSF1R, CD68, CD163, NR4A1, NR4A2, S1PR1
DAM-like	TREM2, TYROBP, APOE, APOC1, LPL, GPNMB, SPP1, CST7, CTSB, CTSD, CTSS, LILRB4, AXL, CSF1R
Autophagy	ULK1, ULK2, ATG13, RB1CC1, ATG101, BECN1, PIK3C3, PIK3R4, ATG14, AMBRA1, WIPI1, WIPI2, ATG3, ATG4A, ATG4B, ATG4C, ATG4D, ATG5, ATG7, ATG10, ATG12, ATG16L1, GABARAP, GABARAPL1, GABARAPL2, MAP1LC3A, MAP1LC3B, SQSTM1, OPTN, TAX1BP1, NBR1, CALCOCO2, TBK1, IRGM, BNIP3, BNIP3L, VPS34, VPS15, UVRAG, RUBCN, ATG9A, ATG2A, ATG2B, TECPR1, EPG5, VPS11, VPS16, VPS18, VPS33A, VPS39, VPS41, RAB7A, RAB11A, RAB5A, RAB5B, RAB5C, RAB7B, SNAP29, STX17, VAMP7, VAMP8, LAMP1, LAMP2, CTSB, CTSD, CTSL, VPS4A, VPS4B, ATG9B, EPN1, DNM2, RUBCNL, KIAA1324
Senescence	TP53, TP73, CDKN1A, CDKN1B, CDKN2A, CDKN2B, CDKN2C, RB1, RB2, E2F1, E2F2, E2F3, CCND1, CCND2, CCND3, CCNE1, CCNE2, CCNA2, CCNB1, CCNB2, CDK1, CDK2, CDK4, CDK6, MDM2, MDM4, GADD45A, GADD45B, GADD45G, PML, CDKN3, CHEK1, CHEK2, ATM, ATR, H2AFX, RAD51, RAD52, RAD9A, PCNA, LMNB1, LMNB2, HMGB1, HMGB2, IL6, IL8, CCL2, CCL5, CXCL1, CXCL2, CXCL3, CXCL5, CXCL8, TNF, IL1A, IL1B, MMP1, MMP3, MMP9, SERPINE1, IGFBP3, IGFBP7, CXCL12, CXCL16, CXCL10, CXCL11, IFNB1, IFNG, TGFBR1, TGFBR2, SMAD2, SMAD3, SMAD4, SMAD7, FOXO1, FOXO3, SIRT1, SIRT6, NAMPT, PARP1, PARP2, CXCR2, CXCR4
Cytoskeleton	ACTB, ACTG1, TUBB, TUBA1A, TUBB2A, TUBB3, VIM, EZR, MSN, RDX, CFL1, CFL2, PFN1, WAS, WASL, ARP2, ARP3, ARPC1A, ARPC1B, ARPC2, ARPC3, ARPC4, ARPC5, DIAPH1, DIAPH2, FMNL1, FMNL2, FMNL3, MYH9, MYH10, MYH14, MYL6, MYL9, TPM1, TPM2, TPM3, TPM4, ADD1, ADD2, ADD3, CORO1A, CORO1B, CORO1C, PLEK, PLEKHA1, PLEKHA2, PLEKHB2, ITGA4, ITGAM, ITGAX, ITGB1, ITGB2, ITGB7, TLN1, TLN2, VCL, PXN, ZYX, RAC1, RAC2, CDC42, RHOA, RHOC, RHOG, DOCK2, DOCK8, ABI1, ABI2, NCK1, NCK2, CRK, CRKL, SOS1, SOS2, RACGAP1, ROCK1, ROCK2, LIMK1, LIMK2, DSTN, PFN2, MYO1E, MYO9B, MYO10, MYO1F, MYO7A, MYO18A, MAP1A, MAP1B, MAP1S, MAP2, MAP4, MAP6, MAP7, MAPK1, MAPK3, MAPK14, MAPK8, MAPK9, MAPK10, PAK1, PAK2, PAK3, PAK4, PAK6, PAK7, KIF1B, KIF5B, KIF11, KIF23, DYNLT1, DYNC1H1, DCTN1, DCTN2, DCTN3, DCTN4, DCTN5, DCTN6, FLNA, FLNB, FLNC, SPTAN1, SPTBN1, SPTBN2, SPTBN4, DBN1, DBN2, FHOD1, FHOD3, LCP1, LCP2
Oxidative Phosphorylation	NDUFA1, NDUFA2, NDUFA3, NDUFA4, NDUFA5, NDUFA6, NDUFA7, NDUFA8, NDUFA9, NDUFA10, NDUFA11, NDUFB1, NDUFB2, NDUFB3, NDUFB4, NDUFB5, NDUFB6, NDUFB7, NDUFB8, NDUFB9, NDUFB10, NDUFB11, NDUFC1, NDUFC2, NDUFS1, NDUFS2, NDUFS3, NDUFS4, NDUFS5, NDUFS6, NDUFS7, NDUFS8, SDHA, SDHB, SDHC, SDHD, UQCRC1, UQCRC2, UQCRFS1, CYC1, COX4I1, COX5A, COX5B, COX6A1, COX6B1, COX6C, COX7A2, COX7B, COX7C, ATP5F1A, ATP5F1B, ATP5F1C, ATP5F1D, ATP5F1E, ATP5MC1, ATP5MC2, ATP5MC3, ATP5ME, ATP5MF, ATP5MG, ATP5PB, ATP5PD, ATP5PF, ATP5PO, ATP5D, ATP5J, ATP5J2, ATP5L, ATP5O
Phagocytosis	FCGR1A, FCGR1B, FCGR1C, FCGR2A, FCGR2B, FCGR3A, FCGR3B, CD36, MSR1, MRC1, ITGAM, ITGB2, LYN, SYK, HCK, LCP2, VAV1, RAC1, RAC2
MHC class II	HLA-DRA, HLA-DRB1, HLA-DRB5, HLA-DPA1, HLA-DPB1, HLA-DQA1, HLA-DQB1, CIITA, CD74, CD86, CD80, CD40, TAP1, TAP2, B2M, LCP2, LY96, IRF1, IRF8, STAT1
Reactive Oxygen Species	SOD1, SOD2, CAT, GPX1, GPX3, GPX4, PRDX1, PRDX2, PRDX3, PRDX4, PRDX5, PRDX6, TXN, TXN2, TXNRD1, TXNRD2, NQO1, HMOX1, GSR, GCLC, GCLM, GSS, GLRX, GLRX2, SRXN1, SESN1, SESN2, KEAP1, NFE2L2
Efferocytosis	MERTK, AXL, TYRO3, GAS6, PROS1, LRP1, CD36, ITGAV, ITGB3, MFGE8, TIMD4, STAB1, STAB2

Table S5: Macrophage functional gene modules. Abbreviations: DAM = disease associated microglia; MHC = major histocompatibility complex.

TF	Family	Mean AD	Mean Ctrl	Δ	Effect size d	CI95 low	CI95 high	P-value	Q-value	Pct active AD	Pct active Ctrl	Δ pct active	Subtype consistency	Subtype mean Δ	Subtype max Δ	n donors with AD	n Ctrl donors	Δ subtype 1	Δ subtype 2	Δ subtype 3
RUNX1	RUNX	0.3039	0.1855	0.1184	0.8927	-0.0485	0.2712	0.2286	0.4604	0.8095	0.1490	0.6605	0.6667	0.0074	0.0462	3	4	0.0462	0.0151	-0.0392
NFIC	Other	0.1365	0.0608	0.0757	1.7075	0.0258	0.1418	0.05714	0.4276	0.8413	0.1433	0.6980	1.0000	0.0666	0.0838	3	4	0.0838	0.0355	0.0804
SFPQ	Other	0.1710	0.0978	0.0732	1.0001	-0.0094	0.1617	0.2286	0.4604	0.6349	0.1805	0.4544	0.3333	0.0068	0.0260	3	4	0.0260	-0.0056	-0.0001
TCF12	Wnt/β-catenin	0.3354	0.2717	0.0637	0.3174	-0.1812	0.2747	0.4000	0.6139	0.6825	0.1719	0.5106	0.3333	-0.1100	0.0337	3	4	-0.1183	0.0337	-0.2454
HMGB2	Other	0.1059	0.0463	0.0596	5.7236	0.0462	0.0721	0.05714	0.4276	0.7143	0.1662	0.5481	1.0000	0.0656	0.0952	3	4	0.0209	0.0952	0.0806
HMGB1	Other	0.0948	0.0472	0.0476	2.1020	0.0215	0.0787	0.05714	0.4276	0.7460	0.1605	0.5856	1.0000	0.0322	0.0429	3	4	0.0276	0.0429	0.0261
GATA5	Other	0.1623	0.1160	0.0463	1.2431	-0.0003	0.0928	0.2286	0.4604	0.4286	0.2178	0.2108	0.6667	0.0075	0.0707	3	4	0.0707	0.0148	-0.0631
CLOCK	Other	0.1103	0.0649	0.0454	1.6401	0.0087	0.0779	0.1143	0.4604	0.4762	0.2092	0.2670	1.0000	0.0253	0.0382	3	4	0.0363	0.0014	0.0382
ZNF600	Other	0.0661	0.0216	0.0444	1.1060	0.0069	0.1074	0.05714	0.4276	0.4286	0.2178	0.2108	1.0000	0.0340	0.0612	3	4	0.0260	0.0612	0.0149
SP100	Other	0.1527	0.1088	0.0439	0.8520	-0.0221	0.0999	0.4000	0.6139	0.4444	0.2149	0.2295	0.3333	-0.0108	0.1094	3	4	0.1094	-0.0146	-0.1270
NFATC2	NFAT (calcium/mech)	0.0958	0.0555	0.0403	1.3706	0.0097	0.0842	0.1143	0.4604	0.7302	0.1633	0.5668	1.0000	0.0309	0.0343	3	4	0.0340	0.0244	0.0343
JUNB	AP-1	0.0926	0.0541	0.0385	1.3417	0.0066	0.0805	0.1143	0.4604	0.6825	0.1719	0.5106	1.0000	0.0256	0.0527	3	4	0.0148	0.0092	0.0527
BPTF	Other	0.1211	0.0828	0.0383	0.8602	-0.0137	0.0925	0.2286	0.4604	0.8889	0.1347	0.7542	0.6667	0.0139	0.0293	3	4	0.0293	0.0157	-0.0032
ZFX	Other	0.0832	0.0460	0.0372	1.4684	0.0084	0.0650	0.1143	0.4604	0.4286	0.2178	0.2108	0.6667	0.0092	0.0248	3	4	-0.0145	0.0172	0.0248
PHF21A	Other	0.1416	0.1045	0.0371	0.7625	-0.0214	0.0899	0.4000	0.6139	0.7619	0.1576	0.6043	0.6667	-0.0053	0.0313	3	4	0.0129	0.0313	-0.0600
RXRA	Other	0.0867	0.0506	0.0360	1.2482	-0.0003	0.0704	0.1143	0.4604	0.7619	0.1576	0.6043	1.0000	0.0230	0.0295	3	4	0.0295	0.0194	0.0202
FOXO3	FOXO (stress/mech)	0.1153	0.0798	0.0355	0.6913	-0.0348	0.0930	0.6286	0.7981	0.6032	0.1862	0.4169	0.6667	0.0324	0.0608	3	4	0.0403	-0.0040	0.0608
STAT2	STAT	0.1428	0.1079	0.0349	0.8936	-0.0200	0.0689	0.4000	0.6139	0.4603	0.2120	0.2483	0.6667	0.0051	0.0479	3	4	0.0393	0.0479	-0.0719
SF1	Other	0.1213	0.0865	0.0348	0.7298	-0.0239	0.0908	0.2286	0.4604	0.8730	0.1375	0.7355	0.3333	0.0040	0.0198	3	4	0.0198	-0.0001	-0.0076
POU2F2	Other	0.0815	0.0481	0.0334	0.8351	-0.0111	0.0882	0.2286	0.4604	0.5873	0.1891	0.3982	0.6667	0.0124	0.0231	3	4	0.0231	-0.0066	0.0208
CHD2	Other	0.1302	0.0979	0.0323	0.6304	-0.0326	0.0921	0.4000	0.6139	0.7619	0.1576	0.6043	0.6667	-0.0002	0.0066	3	4	0.0066	-0.0094	0.0022
ARID3A	Other	0.0784	0.0469	0.0315	2.7641	0.0176	0.0460	0.05714	0.4276	0.7619	0.1576	0.6043	1.0000	0.0331	0.0367	3	4	0.0367	0.0361	0.0264
TFEC	Other	0.1019	0.0714	0.0305	0.7254	-0.0181	0.0855	0.2286	0.4604	0.7778	0.1547	0.6230	0.6667	0.0086	0.0298	3	4	-0.0077	0.0298	0.0037
TCF7L2	Wnt/β-catenin	0.0680	0.0386	0.0294	2.2923	0.0125	0.0468	0.05714	0.4276	0.5714	0.1920	0.3795	1.0000	0.0228	0.0259	3	4	0.0203	0.0259	0.0223
ZNF257	Other	0.0835	0.0541	0.0294	0.4370	-0.0483	0.1227	1.0000	1.0000	0.1270	0.2722	-0.1452	1.0000	0.0275	0.0608	3	4	0.0608	0.0209	0.0007
FLI1	Other	0.1363	0.1072	0.0291	0.4618	-0.0443	0.1011	0.2286	0.4604	0.8413	0.1433	0.6980	0.6667	-0.0107	0.0199	3	4	0.0199	0.0005	-0.0524
ZNF101	Other	0.0736	0.0450	0.0286	4.8271	0.0210	0.0359	0.05714	0.4276	0.4127	0.2206	0.1921	1.0000	0.0299	0.0377	3	4	0.0377	0.0265	0.0254
BHLHE41	Other	0.0708	0.0422	0.0286	1.4844	0.0032	0.0492	0.1143	0.4604	0.6984	0.1691	0.5294	1.0000	0.0237	0.0404	3	4	0.0219	0.0088	0.0404
ZNF41	Other	0.0660	0.0376	0.0284	2.5196	0.0154	0.0437	0.05714	0.4276	0.4921	0.2063	0.2858	1.0000	0.0225	0.0375	3	4	0.0375	0.0157	0.0142
EP300	Other	0.0937	0.0660	0.0277	0.8436	-0.0108	0.0673	0.2286	0.4604	0.8571	0.1404	0.7167	1.0000	0.0071	0.0148	3	4	0.0148	0.0048	0.0018
JUN	AP-1	0.0656	0.0389	0.0267	5.8983	0.0208	0.0322	0.05714	0.4276	0.7937	0.1519	0.6418	1.0000	0.0366	0.0458	3	4	0.0406	0.0458	0.0233
BHLHE40	Other	0.0768	0.0502	0.0266	3.9486	0.0180	0.0344	0.05714	0.4276	0.8254	0.1461	0.6793	1.0000	0.0216	0.0224	3	4	0.0212	0.0212	0.0224
CEBPD	Other	0.0524	0.0262	0.0262	2.2827	0.0148	0.0431	0.05714	0.4276	0.5397	0.1977	0.3420	1.0000	0.0305	0.0470	3	4	0.0068	0.0470	0.0377
ZNF274	Other	0.0814	0.0553	0.0261	3.3011	0.0165	0.0354	0.05714	0.4276	0.6984	0.1691	0.5294	1.0000	0.0194	0.0353	3	4	0.0353	0.0084	0.0144
SPI1	Other	0.0800	0.0539	0.0261	1.1054	-0.0020	0.0550	0.2286	0.4604	0.8571	0.1404	0.7167	1.0000	0.0132	0.0150	3	4	0.0150	0.0111	0.0134
ARNT	Other	0.0763	0.0503	0.0261	1.0459	-0.0027	0.0589	0.2286	0.4604	0.7302	0.1633	0.5668	1.0000	0.0117	0.0179	3	4	0.0179	0.0136	0.0037
KLF6	KLF (shear/mech)	0.0891	0.0631	0.0260	0.9672	-0.0051	0.0579	0.2286	0.4604	0.7460	0.1605	0.5856	0.3333	0.0206	0.0667	3	4	0.0667	-0.0001	-0.0047
MITF	Other	0.1105	0.0846	0.0259	0.6064	-0.0259	0.0767	0.4000	0.6139	0.8413	0.1433	0.6980	0.3333	-0.0025	0.0215	3	4	0.0215	-0.0032	-0.0257
USF2	Other	0.0690	0.0446	0.0244	2.1648	0.0102	0.0368	0.1143	0.4604	0.8730	0.1375	0.7355	1.0000	0.0204	0.0372	3	4	0.0372	0.0121	0.0120
ZSCAN26	Other	0.0981	0.0737	0.0243	1.6440	0.0073	0.0427	0.2286	0.4604	0.4127	0.2206	0.1921	0.6667	0.0108	0.0235	3	4	-0.0030	0.0118	0.0235
HDAC1	Other	0.0878	0.0638	0.0241	0.9046	-0.0094	0.0555	0.2286	0.4604	0.6349	0.1805	0.4544	0.6667	0.0226	0.0509	3	4	-0.0052	0.0509	0.0223
ZNF479	Other	0.0901	0.0662	0.0240	5.2113	0.0189	0.0301	0.05714	0.4276	0.4127	0.2206	0.1921	1.0000	0.0202	0.0486	3	4	0.0111	0.0008	0.0486
CREB5	CREB (cAMP/mech)	0.0742	0.0502	0.0240	1.1055	-0.0011	0.0512	0.2286	0.4604	0.6032	0.1834	0.4198	0.6667	0.0094	0.0265	3	4	0.0109	-0.0093	0.0265
JUND	Other	0.0772	0.0536	0.0236	1.3467	0.0032	0.0452	0.2286	0.4604	0.8571	0.1404	0.7167	1.0000	0.0182	0.0383	3	4	0.0383	0.0065	0.0100
SREBF2	Other	0.0757	0.0526	0.0231	1.1085	-0.0018	0.0480	0.2286	0.4604	0.8889	0.1347	0.7542	1.0000	0.0107	0.0177	3	4	0.0177	0.0064	0.0080
IRF4	Interferon	0.0951	0.0720	0.0231	0.6802	-0.0156	0.0647	0.2286	0.4604	0.5397	0.1977	0.3420	0.3333	0.0129	0.0872	3	4	-0.0263	0.0872	-0.0222
ZNF780A	Other	0.0332	0.0102	0.0230	2.2772	0.0089	0.0354	0.05714	0.4276	0.3492	0.2321	0.1171	1.0000	0.0167	0.0237	3	4	0.0237	0.0181	0.0083
BCLAF1	Other	0.0760	0.0532	0.0228	1.0582	-0.0024	0.0492	0.2286	0.4604	0.8889	0.1347	0.7542	1.0000	0.0109	0.0138	3	4	0.0138	0.0127	0.0064
ELK4	Other	0.0653	0.0425	0.0227	1.3654	0.0007	0.0416	0.1143	0.4604	0.7143	0.1662	0.5481	0.6667	0.0087	0.0149	3	4	-0.0013	0.0125	0.0149

Table S6: Full transcription factor regulon summary comparing AD_ChP and control_ChP macrophages. Δ denotes AD minus control. Subtype consistency indicates the fraction of macrophage subtypes with increased regulon activity in AD.

2 Supplemental Figures

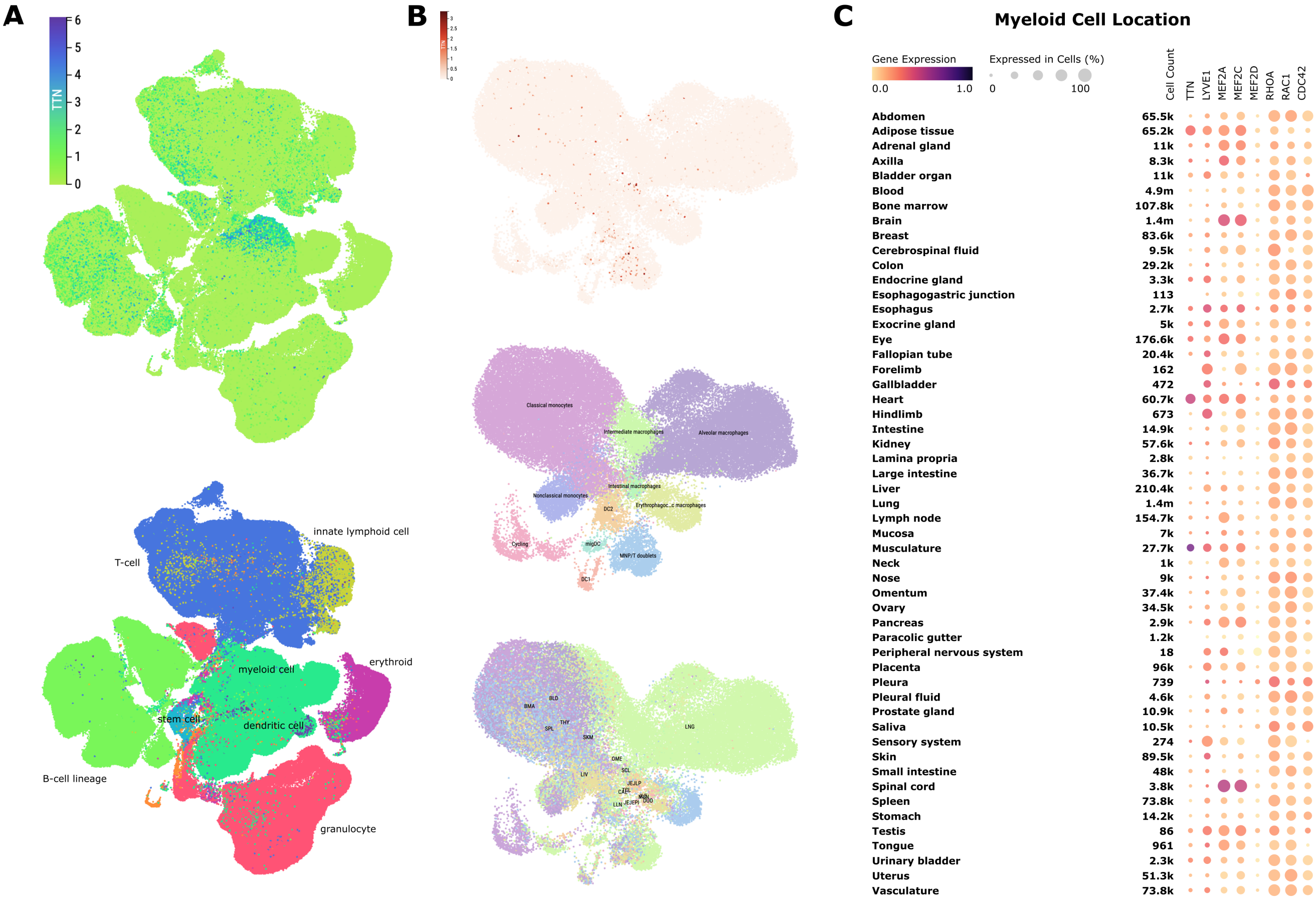

Figure S1: TTN-expression in macrophages in other body tissues. **(A)** Single cell data from the immune cell collection of the Tabula Sapiens [1] reference human cell atlas v2.0 [2], projected onto UMAP coordinates and labeled by TTN expression (top) and immune cell type (bottom). Expression is observed predominantly within the myeloid cell cluster, along with lymphoid subpopulations. **(B)** Single cell data from the cross tissue immune cell atlas [3], labeled by TTN expression (top), immune cell subtype (middle), and organ of residence (bottom). In addition to myeloid lymphocyte doublets, TTN expression is seen in intestinal macrophages. Abbreviations: BLD=blood, BMA=bone marrow, SPL=spleen, THY=thymus, SKM=skeletal muscle, LIV=liver, OME=omentum, LNG=lung, SCL=sigmoid colon, CAE=caecum, JEJLP=jejunum lamina propria, JEJEPI = jejunum epithelium, LLN=lung draining lymph nodes, MLN = mesenteric lymph nodes. **(C)** Expression of TTN and other mechanosensitive genes in myeloid cells by anatomic location, in [2]. In addition to those occupying skeletal muscle and heart, TTN expression in myeloid cells is elevated in adipose tissue

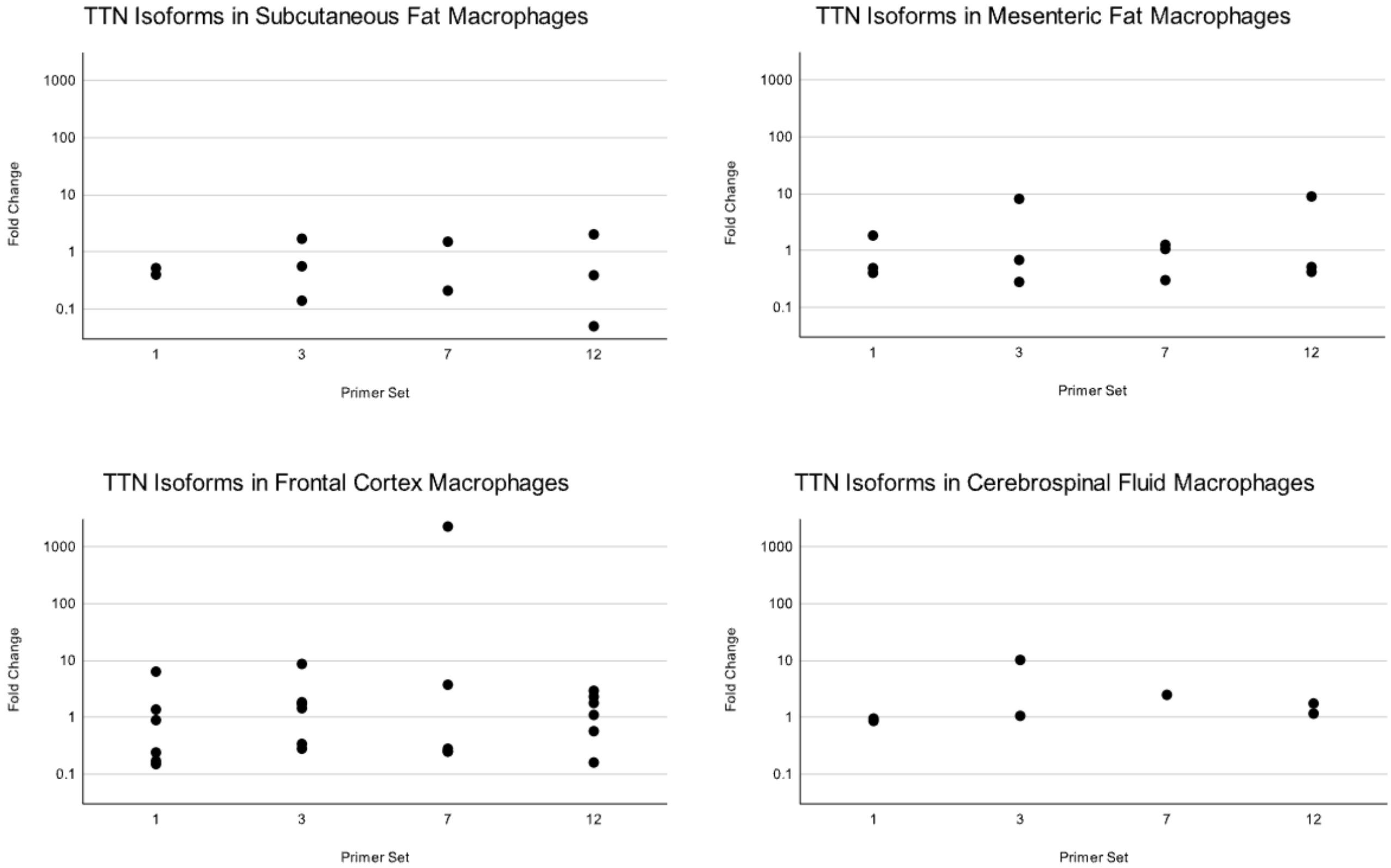

Figure S2: qPCR on all tissues. Macrophages were selected for using CD163 MicroBeads (see Methods). TTN primers 1, 3, 7, and 12 were used to amplify gene expression in human subcutaneous fat, mesenteric fat, frontal cortex, and CSF macrophages. Data points represent fold changes, computed from $\Delta\Delta$Cq, in TTN isoform expression, normalized to GAPDH, in CD163-bead enriched macrophages relative to whole tissue. The x-axis represents different primer sets (1, 3, 7, and 12), and the y-axis shows fold changes (log scale). Each point represents a single donor sample/primer set pair. TTN transcript enrichment is noted in macrophages isolated from frontal cortex, CSF, and, to a lesser degree, mesenteric fat.

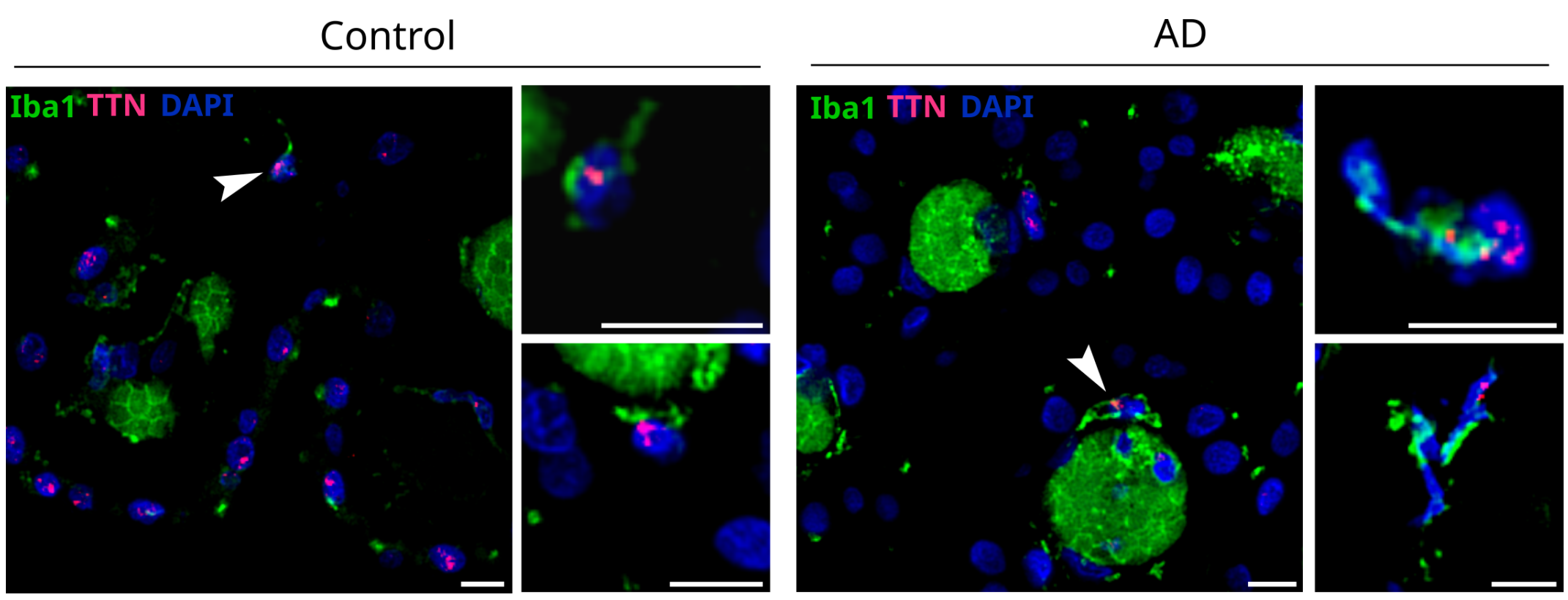

Figure S3: Example micrographs of macrophages in control and Alzheimer's disease choroid plexus used for quantification, stained with Iba1 immunofluorescence (green), RNAscope single molecule FISH probes for TTN RNA (red), and DAPI nuclear stain (blue). All scale bars = 10 µm

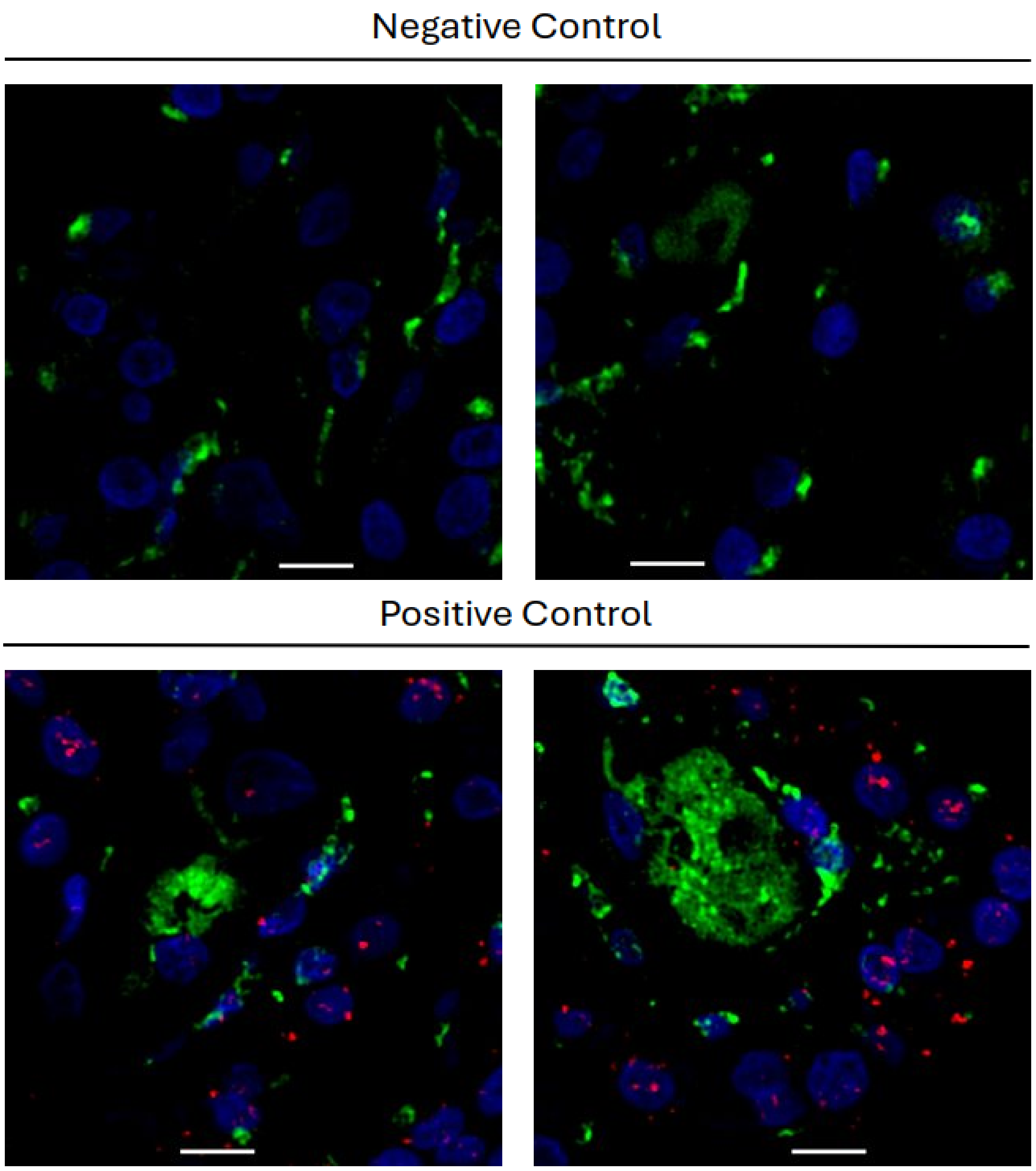

Figure S4: RNAscope Controls on human choroid plexus FFPE tissue. Negative control tissues were incubated with RNAscope 3-plex Negative Control Probe (ACDBio, #320871) for dapB. Positive control received RNAscope 3-plex Positive Control Probe (ACD Bio, #320861) with probes for C1-POL2A. Red = RNAscope probe. Green = Immunofluorescence with Iba1 primary antibody. Scale bars = 10 µm

References (Supplemental Information)

1. THE TABULA SAPIENS CONSORTIUM. The Tabula Sapiens: A multiple-organ, single-cell transcriptomic atlas of humans. *Science* **376,** eabl4896. `https://www.science.org/doi/10.1126/science.abl4896` (May 2022).

2. Consortium, T. T. S. & Quake, S. R. *Tabula Sapiens reveals transcription factor expression, senescence effects, and sex-specific features in cell types from 28 human organs and tissues* en. ISSN: 2692-8205 Pages: 2024.12.03.626516 Section: New Results. Aug. 2025. `https://www.biorxiv.org/content/10.1101/2024.12.03.626516v2`.

3. Domínguez Conde, C. *et al.* Cross-tissue immune cell analysis reveals tissue-specific features in humans. *Science* **376,** eabl5197. `https://www.science.org/doi/10.1126/science.abl5197` (2025) (May 2022).
